# Supplementary material for: Stressors, self-reported overall health, potential protective factors and the workplace well-being of nurses during the COVID-19 pandemic in Switzerland: a longitudinal mixed-methods study protocol
Source: BMJ Open. 2021 Dec 23;11(12):e057021. doi: 10.1136/bmjopen-2021-057021 (PMC8704022; doi:10.1136/bmjopen-2021-057021)
Supplement: Author's manuscript [file bmjopen-2021-057021.draft_revisions.pdf]

BMJ Open

BMJ Open is committed to open peer review. As part of this commitment we make the peer review history of every article we publish publicly available.

When an article is published we post the peer reviewers' comments and the authors' responses online. We also post the versions of the paper that were used during peer review. These are the versions that the peer review comments apply to.

The versions of the paper that follow are the versions that were submitted during the peer review process. They are not the versions of record or the final published versions. They should not be cited or distributed as the published version of this manuscript.

BMJ Open is an open access journal and the full, final, typeset and author-corrected version of record of the manuscript is available on our site with no access controls, subscription charges or pay-per-view fees (http://bmjopen.bmj.com).

If you have any questions on BMJ Open's open peer review process please email info.bmjopen@bmj.com

BMJ Open

Stressors, self-reported overall health, potential protective factors and the workplace well-being of nurses during the COVID-19 pandemic in Switzerland: a longitudinal mixed-methods study protocol

Journal:	*BMJ Open*
Manuscript ID	bmjopen-2021-057021
Article Type:	Protocol
Date Submitted by the Author:	07-Sep-2021

Complete List of Authors:	Ortoleva Bucher, Claudia; University of Applied Sciences Western Switzerland La Source School of Nursing<br>Delmas, Philippe ; University of Applied Sciences Western Switzerland La Source School of Nursing<br>Oulevey Bachmann, Annie; University of Applied Sciences Western Switzerland La Source School of Nursing<br>Gilles, Ingrid; Center for Primary Care and Public Health, Epidemiology and Health Systems

Keywords:	PUBLIC HEALTH, COVID-19, MENTAL HEALTH

SCHOLARONE™
Manuscripts

TITLE PAGE

a) All authors' full names: Claudia ORTOLEVA BUCHER, Philippe DELMAS, Annie OULEVEY BACHMAN, Ingrid GILLES

b) Corresponding author: Claudia Ortoleva Bucher, HES-SO University of Applied Sciences and Arts Western Switzerland, La Source School of Nursing Sciences, Avenue Vinet 30, 1004 Lausanne, Switzerland. ORCID ID: 0000-0002-8411-4181, E-mail: c.ortolevabucher@ecolelasource.ch, Phone: +41 21 556 40 42

c) Claudia ORTOLEVA BUCHER: La Source School of Nursing Sciences, HES-SO University of Applied Sciences and Arts Western Switzerland, Switzerland

Philippe DELMAS: La Source School of Nursing Sciences, HES-SO University of Applied Sciences and Arts Western Switzerland, Switzerland. ORCID ID: 0000-0001-8169-470X

Annie OULEVEY BACHMAN: La Source School of Nursing Sciences, HES-SO University of Applied Sciences and Arts Western Switzerland, Switzerland. ORCID ID: 0000-0001-9537-3587

Ingrid GILLES: Center for Primary Care and Public Health (Unisanté), University of Lausanne, Lausanne, Switzerland. ORCID ID: 0000-0003-2051-4749

d) Keywords: public health, covid-19, mental health

e) No. of words: 4451

Title: Stressors, self-reported overall health, potential protective factors and the workplace well-being of nurses during the COVID-19 pandemic in Switzerland: a longitudinal mixed-methods study protocol

Abstract

Aim: To explore and describe stressors and the resources nurses used to remain healthy during the COVID-19 pandemic.

Background: The COVID-19 pandemic was making a huge impact on Europe's healthcare systems in the spring of 2020, and most predictive models concurred that pandemic waves were in the offing. Most studies adopted a pathogenic approach to the subject; few used a salutogenic approach. These showed, however, that nurses can retain their health despite a pandemic by mobilising generalised resistance resources'. Our study aims to understand how nurses working in Switzerland's hospitals protected their health and workplace well-being during the COVID-19 pandemic by investigating the moderating effects of the health resources they mobilised against the stressors inherent to the situation.

Method: We will use a concurrent mixed-methods panel design with qualitative analyses ancillary to quantitative analyses. Quantitative data will be collected using electronic questionnaires at four time points over two years. Qualitative data will be collected using focus groups. Nurses from Switzerland's two main linguistic regions who had direct, indirect or no contact with COVID-19 patients will be invited to participate. The *a priori* sample size will be at least 3,631 participants at T0 and 1,852 at T4.

Analysis: Longitudinal structural equation modelling and Knowledge Mapping will be used to analyse quantitative and qualitative data, respectively. The results derived from the two data types will then be compared and discussed using a side-by-side approach to determine whether they agree or disagree and how they complement each other to achieve our aims.

Ethics and dissemination: Nurses will receive an electronic informed consent form. The data collected will be stored on a secure server at the authors' institution. This research project was approved by the Human Research Ethics Committee of the Canton of Vaud (2020-02845).

STRENGTHS AND LIMITATIONS OF THIS STUDY

- The use of a salutogenic approach focussing on nurses' resources or protective health factors for dealing with stressful situations.
- The use of a mixed-methods design to enrich the examination of possible links between nurses' resources or protective health factors and workplace well-being (the study's key contribution).
- The use of a longitudinal design to examine changes in our two outcomes over time (exposure to COVID-related stressors and workplace well-being), in exposure to COVID-related stressors, in factors protective of health and in associations between them.
- The potential design adaptations needed due to uncertainties about the ongoing pandemic situation.
- The risks of attrition due to lengthy data collection.

For peer review only - http://bmjopen.bmj.com/site/about/guidelines.xhtml

INTRODUCTION

The COVID-19 pandemic began in China in December 2019. Despite the imposition of drastic confinement measures, Switzerland's healthcare system was very badly affected—in some countries, they were completely overwhelmed [1 2].

Pandemic situations expose nurses to different types of stressors, such as performing unusual tasks in unusual settings (e.g., units specially dedicated to the care of infected patients) and adapting to unusual work shifts. Such exposure has been associated with high levels of psychological distress [3-6] and headaches [7]. During the SARS epidemic, studies reported that between 17.3% and 75.3% of healthcare workers (HCWs) presented with mental health problems [8-12]. Two recent systematic reviews regarding SARS, together with the current literature on COVID-19, have shown that HCWs who worked in COVID-19 units were at a high risk of developing the same psychological symptoms [3 6]. Compared with other HCWs, nurses reported higher stress levels, more psychopathological symptoms and more post-traumatic stress symptoms [13 14]. These problems can persist for up to two years after the end of an epidemic [15]. Such stressful situations generally result in greater cynism vis-à-vis patients, more mistakes and safety issues, poorer communication among nurses and increased costs for healthcare services in terms of both human and financial resources [16 17]. Moreover, nurses are at a higher risk of burnout [17-19]. Most studies in this area have taken a pathogenic approach, focussing on identifying diseases, symptoms and risk factors [3 4]. Few studies have adopted a salutogenic approach focussing on well-being and identifying the health resources mobilised by HCWs to help them cope with their contextual stressors. Salutogenesis is "a scholarly orientation focusing attention on the study of the origins of health and assets for health, contra the origins of disease and risk factors" [20]. It considers health as a continuum from optimal health to disease. When developing preventive actions, and instead of simply considering risk factors, salutogenesis also aims to highlight the so-called 'factors protective of health' or the 'generalised health resources' that actively support health [20]. Studies adopting this approach have shown that nurses can retain their health in such situations by mobilising 'generalised resistance resources' [20]. For example, two preliminary studies conducted in Belgium [21] and Switzerland's French-speaking region [22] have shown that the pandemic's impact has not been entirely negative and that nurses managed to find the resources to meet the challenges they faced. Gaining clearer insight into how nurses maintained their health in the face of a pandemic is essential. The salutogenic perspective has rarely been explored in a pandemic context despite its real-world applications and may provide blueprints for developing preventive interventions aimed at maintaining HCWs' health and workplace well-being during times of pandemic. Therefore, this research project will adopt a salutogenic perspective.

Most studies conducted on this topic to date have been atheoretical [3 4]. Accordingly, the relationships between concepts have varied widely. Depression, for example, has been considered a predictor and an outcome [3 4]. To describe, explain and understand the nature and meaning of phenomena, and to develop interventions in the health field, research should be based on established models [23]. Our theoretical framework will be the Neuman Systems Model (NSM), considered a salutogenic model according to the criteria of both Lindström and Eriksson [24] and Mittelmark and Bauer [20]. The NSM considers health to be in a dynamic equilibrium in which human beings can retain, attain and retain stability, and in which, perceived wellness is viewed as a manifestation of health [25]. Stressors are considered neutral *a priori*; their impact depends on circumstances, the moment, and the individual's perception of them and ability to cope with them ('eustress' and 'stress') [25]. An individual's ability to retain health and well-being is subject to two mechanisms: reducing exposure to stressors and activating factors protective of health. These protective factors may be physiological, psychological, socio-cultural, developmental or spiritual, and they may act as moderators between stressors and health [25 26]. Finally, an individual's ability to retain their health is accordion-like: when strengthened by primary preventive interventions, it can develop and become more effective. The NSM's theoretical framework seems well suited for our study because it enables an exploration of: 1) the stressors to which HCWs' health is exposed; 2) their relationships with HCWs' overall health and well-being over time; and 3) the mechanisms used to stay healthy despite this exposure (Figure 1).

Another limitation identified in studies exploring the impact of the COVID-19 pandemic or other epidemics (SARS or MERS) on HCWs' health is their cross-sectional design and/or post-pandemic research design [3 4]. These allowed for no examination of changes in nurses' health over time during the pandemic episode, nor for any determination of causality between exposure and outcomes. Longitudinal studies are needed to establish causal relationships between stressors, moderators and outcomes in the context of COVID-19 [27]. To the best of our knowledge, there are only two ongoing longitudinal studies analyzing nurses' health during COVID-19 [28 29]. In Switzerland, Fuchs *et al.*, [29] conducted a longitudinal mixed-methods study, which started in April and was planned to end in December 2020, to analyse HCWs' mental health using a pathogenic perspective (outcomes include symptoms such as anxiety, depression and PTSD). In Canada, Richardson *et al*. have been monitoring HCWs for 18 months to measure their well-being and levels of distress [28]. Our proposed longitudinal mixed-methods study using a salutogenic approach would complement these two timely studies.

Because this unprecedented pandemic situation is ongoing, with no end in sight [27], our research project based on the NSM has the following objectives:

- i) to understand how nurses working in Switzerland's hospitals retained their health and workplace well-being throughout the COVID-19 pandemic (mixed-methods);
- ii) to explore the stressors experienced by nurses throughout the months of the pandemic (qualitative methods);
- iii) to measure changes in their experience of these stressors and in any possible repercussions on their overall health and workplace well-being during the pandemic (quantitative methods);
- iv) to explore and describe the resources used to remain healthy during the pandemic (quantitative and qualitative methods).

METHODS AND ANALYSIS

Study design overview

We will use a concurrent mixed-methods panel design. Quantitative analyses [30] will use data from four successive waves of self-reporting questionnaires at six-month intervals (T0, baseline; T1, at six months; T2, at 12 months; and T3 at 18 months). This survey started in March 2021 in French-speaking Switzerland and will start in September 2021 in German-speaking Switzerland (Figure 2). Qualitative interviews will take place in parallel using two focus group (FG) phases (in September 2021 and 2022) in the respective linguistic regions.

Both methodological parts are detailed below, and several reasons led to their selection. Concerning the mixed-methods approach, it is recommended that multi-faceted research questions use more than one method of investigation [31], and, in our case, understanding stressors requires just such in-depth exploration. A longitudinal design will be necessary to evaluate changes over time [32]. According to Wang *et al*. [32], at least three time points are necessary to provide a robust picture of changes in socio-psychological variables and how they relate to one another. Based on the most common longitudinal wave time-point intervals reported in the literature (6–12-months) (2) and our own research experience (4, 5), we chose a 6-month interval.

Quantitative part

A self-administered electronic survey covering socio-demographic and psychological questionnaire items will be sent to nurses at 15 hospitals and clinics in the French- and German-speaking regions of Switzerland by email. No sampling strategy will be applied; every eligible nurse will be contacted. A

communication strategy to encourage participation has been developed (emails announcing the study, reminders). The data collection phase at each time point (T0 to T3) will last one month.

Population and sampling

The population includes all the nurses working in our partnering healthcare institutions at baseline, regardless of whether they have had direct, indirect or no contact with patients infected with COVID-19. Fifteen institutions have already agreed to facilitate the study, making our potential population 5,187 nurses working in different hospitals in Switzerland's German- and French-speaking regions.

Inclusion criteria at baseline are: (i) having a long-term (not temporary) contract at the institution selected; (ii) working at least 50% of a full-time equivalent position; (iii) being able to read and understand French or German; and (iv) having signed the written informed consent form. Exclusion criteria at baseline are: (i) being a nurse manager; (ii) not working at the institution during the COVID-19 crisis; and (iii) being a student nurse during the COVID-19 crisis.

Recruitment targets

We used previous relevant studies to estimate participation and attrition rates. A recent Swiss survey of HCWs' overall health had a response rate of 37% [22]. Concerning attrition rates, Niu *et al*. [33] expect 20% in their study of nurse sleep quality and Kovner *et al*. [34] reported a 30% attrition rate in their panel study of nurse turnover. The Swiss Household Panel survey recorded attrition rates from 13% to 18% [35]. We therefore estimate a 20% attrition rate at each data collection time point. Thus, we expect our accessible population of 5,187 nurses at T0 to fall to 3,631 at T1 (80% of T0 staff), 2,905 at T2 (80% of T1 staff), 2,324 at T3 (64% of T1 staff), with a final sample of 1,852 at T4 (51% of T1 staff).

Measurement

The questionnaire is composed of the following scales:

The Perceived Stress Scale (PSS) [36], in validated French and German versions [37 38], is a 10-item scale to evaluate the degree to which life situations are generally perceived as threatening, that is, as unpredictable, uncontrollable and painful [39].

The Psychological Well-Being Scale, developed by Diener *et al*. [40] and adapted for the workplace by Fisher [41], in validated French and German versions [42-44], is an 8-item scale to evaluate workplace well-being and measures self-perceived functioning in areas such as self-esteem, purpose and relationships.

For peer review only - http://bmjopen.bmj.com/site/about/guidelines.xhtml

The World Health Organization Quality of Life – BREF (WHOQOL-BREF) [45], in validated French and German versions [46 47], is a 26-item short version of the 100-item WHOQOL scale covering four domains—physical health, mental well-being, social relations and work environment—that measure self-perceived quality of life.

The Brief-COPE [48], in validated French and German versions [49 50], is a 28-item scale evaluating several coping strategies.

The Post-traumatic Growth Inventory-Short Form (PTGI-SF) [51], in validated French and German versions [52-54], is a 10-item inventory evaluating post-traumatic growth, that is, positive psychological change experienced following a traumatic event.

The 10-item Connor–Davidson Resilience Scale (CD-RISC®) [55], in validated French and German versions [55 56], measures a person's ability to bounce back when confronted with the difficulties that may arise in life.

The Multidimensional Scale of Perceived Social Support (MSPSS) [57], in validated French and German versions [58-60], is a 12-item scale measuring perceived social support from family, friends and significant others.

The Copenhagen Psychosocial Questionnaire (COPSOQ) [61], in validated French and German versions [62 63], is a comprehensive tool for assessing psychosocial risk in the workplace through 24 core dimensions covering four aspects of work: work environment, health, well-being and personality. We will use three of these dimensions: dimensions of social support from colleagues, dimensions of social support from supervisors and dimensions of satisfaction with job quality.

Professional identification will be measured using the single-item measure of social identification proposed by Postmes *et al*. [64], which can be adapted to any social group, including professional ones. Its simple wording ("I identify with [target group]" reduces interpretation bias and translation issues; its 7-point rating scale ranges from 1 = fully disagree to 7 = fully agree.

Socio-demographic variables. Information regarding gender, age, family situation, and number of children, employment rate, years since last diploma, years of experience in current department, level of exposure to COVID-19 (nurses working in COVID-19 care units; nurses working in institutions that admitted infected patients, but not in COVID-19 units; nurses working in institutions that did not admit infected patients) and continuing professional development program completed will be collected. As suggested by Aiken *et al*. [65], hospitals' organisational indicators (e.g. nurse–patient ratios, work schedules, average time spent with patients per shift) will be collected at each measurement point.

For peer review only - http://bmjopen.bmj.com/site/about/guidelines.xhtml

Quantitative data analyses

Standard data-quality checks will be performed to remove unreliable values from the database, and descriptive statistics will be computed to qualify those data. Associations between perceived stress and well-being will subsequently be modelled using mixed-effects regressions. Random effects are necessary to model the data's longitudinal structure. Linear models will be the default choice, but we will not rule out resorting to data manipulation (e.g. dichotomisation) or alternative models (e.g. logistic models) if the data's structure requires it. The moderating effects of post-traumatic growth, resilience, perceived support, coping strategies, job satisfaction and professional identity will be evaluated by entering the estimated interactions between these variables and perceived stress into the regression models. A frequentist probability approach will be used for all our analyses. All calculations will be run on the latest available stable version of R software. Statistical significance will be set at $\alpha = 0.95$.

Qualitative part

We will use a knowledge mapping approach [66]. Mind mapping is a useful graphical format for representing key themes raised during FGs. It can help stimulate and galvanise discussions and keep them on track, enhance transparency and group ownership of the data analysis process, and enable a rapid dynamic to develop between data collection and feedback [67]. This pragmatic approach is considerably faster than traditional methods used for analysing FGs, but it produces broadly similar results [67]. Participants are actively involved in interpreting the resulting mind maps [68]. The advantages of mapping include its 'free form' and unconstrained structure, meaning there are no limits to the ideas and links that can be made and no need to retain an ideal structure or format. Mapping thus promotes creative thinking and encourages brainstorming. The three steps to structuring a knowledge mapping approach are: (1) developing interview guidelines, (2) conducting FG interviews and (3) analysis and conclusion [66].

Population

The target population will consist exclusively of nurses who respond to the questionnaire at T0 and indicate their interest in participating in the study's future stages by giving their written informed consent at that time. The interested nurses in each linguistic region will be divided into three groups according to their degree of contact with COVID-19 patients: 1) nurses in direct contact with infected patients (worked in COVID-19 care units); 2) nurses in indirect contact because their institution admitted infected patients, but they did not work in COVID-19 care units; 3) nurses with no contact because their institutions did not admit infected patients.

For peer review only - http://bmjopen.bmj.com/site/about/guidelines.xhtml

Sampling and recruitment targets

The research team will select three samples of nurses to participate in FGs in each of the two linguistic regions 12 months apart. Nurses will be allowed to participate in the FGs during their paid work hours. If there are too many volunteers, participants will be selected using a convenience sampling method based on socio-demographic characteristics such as age, their institution's geographical location, whether their COVID-19 experience had been on their usual ward, and to foster heterogeneity among the FG participants. If there has been attrition in the FGs at T3, groups will be completed with new participants selected in the same way.

Data collection

Investigators began by developing a semi-structured interview guide based on the NSM and tested it on two nurses not participating in the study. Several pathways will be explored: lived experiences and stressors, health, well-being at work, as well as the evolution of these components over time and the resources mobilised to retain them.

FG discussion will last about 1.5 hours and will be held by videoconference that facilitates mutual trust and ensure confidentiality. Participants will be formally asked for their consent before conversations are recorded. A moderator (a senior researcher) will lead each FG, assisted by a scientific collaborator. Both will be trained beforehand and will receive precise guidelines to ensure standardised procedures. All the topics in the interview guide will be discussed.

A knowledge map of each topic discussed will be drawn on a flip chart, resulting in a synthesis formed by a complex network of representations in text: "A knowledge map is the visual display of captured information and relationships […]" [69]. Central concepts within each topic will be arranged in relation to one another, and recurring concepts will be highlighted. Before moving from one topic to the next, the moderator will ensure that every input has been documented and that participants validate the map to ensure that the researchers have properly understood each point as they have been expressed [66]. The knowledge maps will also be considered as protocols for the FG discussions.

Qualitative data analysis

Qualitative analysis will begin during the FG sessions when participant feedback confirms that each map fully summarises each topic's discussion. Knowledge maps will then be digitised and merged by interview topic. They will then be translated into English to enable the complete integration of the different German and French language maps. The integrated maps in English will then be discussed,

For peer review only - http://bmjopen.bmj.com/site/about/guidelines.xhtml

and the qualitative research team will cluster themes. Each cluster will be described with regards to the study's aims.

To ensure rigour in the qualitative data collection process, the investigators will maintain a logbook of methodological, theoretical and personal notes.

Integrating quantitative and qualitative data

Data integration will occur at a workshop bringing together the qualitative and quantitative research teams. This will be held in English to facilitate communication among the participants and because our results will be published in English language scientific journals. We will carefully identify any divergences or inconsistencies between the quantitative and qualitative results and attempt to explain them.

During this step, the qualitative data in English will be combined with the quantitative data. The results derived from both types of data, which will have been presented and analysed separately beforehand, will then be compared and discussed using a side-by-side approach [30] to determine whether they converge or diverge and how they complement one another to answer our research questions and study objectives.

Patient and public involvement

Patients and/or the public were involved in the design, conduct, reporting or dissemination plans of this research. Refer to the Methods and analysis section for further details.

Study status

Participant recruitment for the survey's first round (T0) began in March 2021 in French-speaking Switzerland. Subsequent rounds are planned for September 2021 (T1), March 2022 (T2) and September 2022 (T3). Participation in German-speaking Switzerland will occur with a 6-month delay: T0 in September 2021, T1 in March 2022, T2 in September 2022 and T3 in March 2023. This time lag will be used to collect qualitative data. Thus, the FGs for French- and German-speaking Switzerland are scheduled for September 2021 and September 2022, respectively.

Ethics and dissemination

This project was approved by the Human Research Ethics Committee of the Canton of Vaud (project no. 2020-02845). Each partner institution will designate a reference person. The research team will

For peer review only - http://bmjopen.bmj.com/site/about/guidelines.xhtml

send that person an anonymous link to the online questionnaire and an information letter and a standard study invitation email to be adapted for each institution.

At T0, each reference person will send out their adapted information email to their institution's potential participants (specifying that the study is longitudinal and includes four waves). These nurses will be free to accept or decline participation. At the end of the questionnaire, participants who wish to join in the next waves and FGs will be asked to provide an email address so the research team can contact them directly.

Participants will be able to withdraw from the study at any time. Email addresses will be kept by the research team member responsible for the logistical aspects of data collection (sending invitation emails and reminders). Other research team members will not have access to this information. Nominative information (e.g. emails) will be deleted at the end of the study.

Participation in both the quantitative and qualitative parts of this study will be voluntary—nurses will be under no obligation. Electronic 'clicked' consent will be collected from all participants in the quantitative part on a form stating that their data may be used and shared with other researchers in an anonymised form and that they will not be identified nominatively in any outputs. All participants will have a unique anonymous identifier, and only this identifier will appear in the datasets. Separate files with personal details will be kept securely, will not be shared with other researchers and will be deleted on completion of the project. At least 72 hours before collecting qualitative data in FGs, participants will receive an information letter and a consent form for signature. Recordings will not be shared, and transcriptions will be anonymised. Personal data enabling the identification of participants will be kept separate from survey responses and will be deleted when data collection is complete. Any data to be made public will be entirely anonymous.

Nurses will be under no obligation to answer any or all of the questions, and they will be free to interrupt questionnaires at any time. In this event, answers already provided will not be registered.

Results will be disseminated in several ways. The first persons to benefit from this research will be the participants themselves. A document containing the study's highlights will be prepared specifically for them and made available on our partner institutions' websites or on demand. These institutions will hold conferences open to the public. Scientific publications will be addressed to the French-, English- and German-speaking scientific communities (e.g. *BMC Health and Services Research, Journal of Clinical Nursing, International Journal of Nursing Research, Recherche en Soins Infirmiers, etc.*). Results will also be disseminated at international and local conferences. Finally, a research report containing the study's key findings will be produced for the libraries of the different partnering institutions and for the funding partner.

For peer review only - http://bmjopen.bmj.com/site/about/guidelines.xhtml

Study significance

We believe that this study will make a notable contribution to advancing scientific knowledge on HCWs' health and workplace well-being during pandemics, particularly on the following aspects: a) analysing changes in the effects of various potential factors protective of nursing health and workplace well-being; b) developing knowledge using an established theoretical framework in a novel context; and c) guiding the development of new preventive interventions to support nurses in times of pandemic. Several elements are worthy of note regarding the potential benefits of this study for our institutional field partners. Study findings could be integrated into quality improvement processes at participating institutions and could inform the development of support programmes there for preventing disease and promoting health among their HCWs.

Embedding the NSM's theoretical framework in a salutogenic approach [25] will provide us with an essential insight into what protects nurses' overall health and workplace well-being during a pandemic such as the one we are living through. It will shed light on the different stressors that they are exposed to. This is a vital step towards developing preventive interventions geared both to mobilising factors protective of health and to reducing exposure to risk factors. Such interventions would support nurses' overall health and workplace well-being.

Broader impacts

Several elements are worthy of note regarding the potential benefits of this study for our institutional partners. The findings could be integrated into their quality improvement processes and could inform the development of support programmes for preventing disease and promoting health among their staff, particularly nurses.

The results of this research could also be integrated into undergraduate nursing curricula or even continuing professional development programmes at partnering institutions or in healthcare management education programmes in Switzerland. More broadly, they will also support the healthcare system's performance when faced with crises of a similar type.

Author contributions

All authors contributed equally to the conceptualization of the study. All authors are involved in the data recruitment. IG hosted the survey and all authors are involved in the data analyses. All authors significantly contributed to the writing of the manuscript. The manuscript was reviewed and edited prior to submission, and all authors agreed on the final version.

Competing interests (for all authors):

For peer review only - http://bmjopen.bmj.com/site/about/guidelines.xhtml

The authors have no conflict of interest to declare.

Funding

The study is supported by the Swiss National Science Fundation (10001C_201137/1).

References

1. Remuzzi A, Remuzzi G. COVID-19 and Italy: what next? *The Lancet* 2020;395(10231):1225-28. doi: 10.1016/S0140-6736(20)30627-9

2. Italian Society of Anesthesia RaICS. Clinical ethics recommendations for the allocation of intensive care treatment, in exceptional, resource-limited circumstances 2020 [Available from: http://www.siaarti.it/SiteAssets/ News/COVID19%20-%20documenti%20SIAARTI/SIAARTI%20-% 20Covid-19%20-%20Clinical%20Ethics%20Reccomendations.pdf accessed 02.09.2020.

3. Preti E, Di Mattei V, Perego G, et al. The psychological impact of epidemic and pandemic outbreaks on healthcare workers: rapid review of the evidence. *Current Psychiatry Reports* 2020;22(8):43. doi: 10.1007/s11920-020-01166-z

4. Stuijfzand S, Deforges C, Sandoz V, et al. Psychological impact of an epidemic/pandemic on the mental health of healthcare professionals: a rapid review. *BMC Public Health* 2020;20(1):1230. doi: 10.1186/s12889-020-09322-z

5. Pappa S, Ntella V, Giannakas T, et al. Prevalence of depression, anxiety, and insomnia among healthcare workers during the COVID-19 pandemic: A systematic review and meta-analysis. *Brain Behav Immun* 2020;88:901-07. doi: 10.1016/j.bbi.2020.05.026 [published Online First: 2020/05/22]

6. Rajkumar RP. COVID-19 and mental health: A review of the existing literature. *Asian Journal of Psychiatry* 2020;52:102066. doi: https://doi.org/10.1016/j.ajp.2020.102066

7. Chew NWS, Lee GKH, Tan BYQ, et al. A multinational, multicentre study on the psychological outcomes and associated physical symptoms amongst healthcare workers during COVID-19 outbreak. *Brain Behav Immun* 2020;88:559-65. doi: 10.1016/j.bbi.2020.04.049 [published Online First: 2020/04/25]

8. Chong M-Y, Wang W-C, Hsieh W-C, et al. Psychological impact of severe acute respiratory syndrome on health workers in a tertiary hospital. *British Journal of Psychiatry* 2004;185(2):127-33. doi: 10.1192/bjp.185.2.127 [published Online First: 2018/01/02]

9. Goulia P, Mantas C, Dimitroula D, et al. General hospital staff worries, perceived sufficiency of information and associated psychological distress during the A/H1N1 influenza pandemic. *BMC Infectious Diseases* 2010;10(1):322. doi: 10.1186/1471-2334-10-322

10. Lu YC, Shu BC, Chang YY, et al. The mental health of hospital workers dealing with severe acute respiratory syndrome. *Psychotherapy and Psychosomatics* 2006;75(6):370-75. doi: 10.1159/000095443

11. Nickell LA, Crighton EJ, Tracy CS, et al. Psychosocial effects of SARS on hospital staff: survey of a large tertiary care institution. *Canadian Medical Association Journal* 2004;170(5):793. doi: 10.1503/cmaj.1031077

12. Tam CWC, Pang EPF, Lam LCW, et al. Severe acute respiratory syndrome (SARS) in Hong Kong in 2003: stress and psychological impact among frontline healthcare workers. *Psychological*

For peer review only - http://bmjopen.bmj.com/site/about/guidelines.xhtml

Medicine 2004;34(7):1197-204. doi: 10.1017/S0033291704002247 [published Online First: 2004/10/21]

13. Matsuishi K, Kawazoe A, Imai H, et al. Psychological impact of the pandemic (H1N1) 2009 on general hospital workers in Kobe. *Psychiatry and Clinical Neurosciences* 2012;66(4):353-60. doi: 10.1111/j.1440-1819.2012.02336.x
14. Lai J, Ma S, Wang Y, et al. Factors associated with mental health outcomes among health care workers exposed to Coronavirus Disease 2019. *JAMA Netw Open* 2020;3(3):e203976. doi: 10.1001/jamanetworkopen.2020.3976 [published Online First: 2020/03/24]
15. Maunder RG, Lancee WJ, Balderson KE, et al. Long-term psychological and occupational effects of providing hospital healthcare during SARS outbreak. *Emerging Infectious Disease journal* 2006;12(12):1924. doi: 10.3201/eid1212.060584
16. Lamiani G, Dordoni P, Argentero P. Value congruence and depressive symptoms among critical care clinicians: The mediating role of moral distress. *Stress Health* 2018;34(1):135-42. doi: 10.1002/smi.2769 [published Online First: 2017/07/01]
17. Austin CL, Saylor R, Finley PJ. Moral distress in physicians and nurses: Impact on professional quality of life and turnover. *Psychol Trauma* 2017;9(4):399-406. doi: 10.1037/tra0000201 [published Online First: 2016/11/01]
18. Christodoulou-Fella M, Middleton N, Papathanassoglou EDE, et al. Exploration of the association between nurses' moral distress and secondary traumatic stress syndrome: Implications for patient safety in mental health services. *Biomed Res Int* 2017;2017:1908712. doi: 10.1155/2017/1908712 [published Online First: 2017/12/07]
19. Papathanassoglou ED, Karanikola MN, Kalafati M, et al. Professional autonomy, collaboration with physicians, and moral distress among European intensive care nurses. *Am J Crit Care* 2012;21(2):e41-52. doi: 10.4037/ajcc2012205 [published Online First: 2012/03/03]
20. Mittelmark M, Bauer G. The Meaning of Salutogenesis. In: Mittelmark M, Sagy S, Eriksson M, et al., eds. The Handbook of Salutogenesis. Cham: Springer 2017.
21. Lecocq D, Delmas P, Antonini M, et al. Comparing feeling of competence regarding humanistic caring in Belgian nurses and nursing students: A comparative cross-sectional study conducted in a French Belgian teaching hospital. *Nursing open* 2020;8(1):104-14. doi: 10.1002/nop2.608
22. Antonini, M. , Perriraz M, Delmas P, et al. Nurses and healthcare assistants' views of their institution's response to first wave of COVID-19 pandemic and their intentions to remain in their current workplace: a first glance at the ProH-COVID project *Bulletin of the Swiss Sociological Association* 2020;157:20-25.
23. Fawcett J, Desanto-Madeya S. Contemporary nursing knowledge : analysis and evaluation of nursing models and theories (3rd ed.). . Philadelphia: F. A. Davis Company 2013.
24. Lindström B, Eriksson M. La salutogenèse : petit guide pour promouvoir la santé. Québec: PUL 2012.
25. Neuman B, Fawcett J. The Neuman Systems Model (5th ed.). Boston: Pearson Education 2011.
26. Gigliotti E. New advances in the use of Neuman's Lines of Defense and Resistance in Quantitative Research. *Nurs Sci Q* 2012;25(4):336-40. doi: 10.1177/0894318412457054
27. Flynn R, Riches E, Reid G, et al. Rapid review of the impact of COVID-19 on mental health. Edinburgh: Public Health Scotland, 2020.
28. Richardson D. Moral distress and well-being in health care workers during COVID-19 pandemic: A longitudinal study Canada: Canadian Psychological Association; 2020 [Available from:

For peer review only - http://bmjopen.bmj.com/site/about/guidelines.xhtml

https://cpa.ca/abstract/moral-distress-and-well-being-of-health-care-workers-during-the-covid-19-pandemic-a-longitudinal-study/ accessed 13.09.2020.

29. Fuchs A, Abegglen S, Berger-Estilita J, et al. Distress and resilience of healthcare professionals during the COVID-19 pandemic (DARVID): study protocol for a mixed-methods research project. *BMJ Open* 2020;10(7):e039832. doi: 10.1136/bmjopen-2020-039832 [published Online First: 2020/08/02]

30. Creswell J, Creswell J. Research design: Qualitative, quantitative, and mixed methods approaches. Los Angeles: Sage Publications 2018.

31. Creswell J, Plano CV. Designing and Conducting Mixed Methods Research, 3rd ed. . Thousand Oaks, CA: Sage Publications Inc. 2013.

32. Wang M, Beal DJ, Chan D, et al. Longitudinal Research: A Panel Discussion on Conceptual Issues, Research Design, and Statistical Techniques. *Work, Aging and Retirement* 2016;3(1):1-24. doi: 10.1093/workar/waw033

33. Niu SF, Miao NF, Liao YM, et al. Sleep quality associated with different work schedules: a longitudinal study of nursing staff. *Biol Res Nurs* 2017;19(4):375-81. doi: 10.1177/1099800417695483 [published Online First: 2017/06/20]

34. Kovner CT, Djukic M, Fatehi FK, et al. Estimating and preventing hospital internal turnover of newly licensed nurses: A panel survey. *Int J Nurs Stud* 2016;60:251-62. doi: 10.1016/j.ijnurstu.2016.05.003 [published Online First: 2016/06/15]

35. Voorpostel M, Tillmann R, Lebert F, et al. Swiss Household Panel Userguide (1999-2016), Wave 18, December 2017. Lausanne: FORS, 2017.

36. Cohen S, Kamarck T, Mermelstein R. A global measure of perceived stress. *J Health Soc Behav* 1983;24(4):385-96. [published Online First: 1983/12/01]

37. Bellinghausen L, Collange J, Botella M, et al. [Factorial validation of the French scale for perceived stress in the workplace]. *Sante Publique* 2009;21(4):365-73. [published Online First: 2010/01/28]

38. Klein EM, Brähler E, Dreier M, et al. The German version of the Perceived Stress Scale - psychometric characteristics in a representative German community sample. *BMC Psychiatry* 2016;16:159. doi: 10.1186/s12888-016-0875-9 [published Online First: 2016/05/25]

39. Quintard B. Du stress objectif au stress perçu. In: Bruchon-Schweitzer M, Dantzer R, eds. Instroduction à la psychologie de la santé. Paris: PUF 1994.

40. Diener E, Wirtz D, Tov W, et al. New well-being measures: short scales to assess flourishing and positive and negative feelings. *Social Indicators Research* 2010;97(2):143-56. doi: 10.1007/s11205-009-9493-y

41. Fisher CD. Conceptualizing and measuring wellbeing at work. *Wellbeing* 2014:1-25. doi: doi:10.1002/9781118539415.wbwell018

10.1002/9781118539415.wbwell018

42. Villieux A, Sovet L, Jung S-C, et al. Psychological flourishing: Validation of the French version of the Flourishing Scale and exploration of its relationships with personality traits. *Personality and Individual Differences* 2016;88:1-5. doi: 10.1016/j.paid.2015.08.027

43. Giuntoli L, Ceccarini F, Sica C, et al. Validation of the Italian versions of the Flourishing Scale and of the Scale of Positive and Negative Experience. *SAGE Open* 2017;7(1):2158244016682293. doi: 10.1177/2158244016682293

For peer review only - http://bmjopen.bmj.com/site/about/guidelines.xhtml

44. Esch T, Jose G, Gimpel C, et al. [The Flourishing Scale (FS) by Diener et al. is now available in an authorized German version (FS-D): application in mind-body medicine]. *Forsch Komplementmed* 2013;20(4):267-75. doi: 10.1159/000354414 [published Online First: 2013/09/14]

45. Skevington SM, Lotfy M, O'Connell KA. The World Health Organization's WHOQOL-BREF quality of life assessment: psychometric properties and results of the international field trial. A report from the WHOQOL group. *Qual Life Res* 2004;13(2):299-310. doi: 10.1023/b:Qure.0000018486.91360.00 [published Online First: 2004/04/17]

46. Leplège A, Debout C. Mesure de la qualité de vie et science des soins infirmiers. *Recherche en soins infirmiers* 2007;88(1):18-24. doi: 10.3917/rsi.088.0018

47. Angermeyer MC, Kilian R, Matschinger H. WHOQOL - 100 und WHOQOL - BREF: Handbuch für die deutschsprachige Version der WHO-Instrumente zur Erfassung von Lebensqualität. Göttingen: Hogrefe 2000.

48. Carver CS, Scheier MF, Weintraub JK. Assessing coping strategies: a theoretically based approach. *J Pers Soc Psychol* 1989;56(2):267-83. doi: 10.1037//0022-3514.56.2.267 [published Online First: 1989/02/01]

49. Muller L, Spitz E. [Multidimensional assessment of coping: validation of the Brief COPE among French population]. *Encephale* 2003;29(6):507-18. [published Online First: 2004/03/19]

50. Knoll N, Rieckmann N, Schwarzer R. Coping as a mediator between personality and stress outcomes: a longitudinal study with cataract surgery patients. *European Journal of Personality* 2005;19(3):229-47. doi: 10.1002/per.546

51. Tedeschi RG, Calhoun LG. The posttraumatic growth inventory: Measuring the positive legacy of trauma. *Journal of Traumatic Stress* 1996;9(3):455-71. doi: 10.1002/jts.2490090305

52. Lelorain S, Bonnaud-Antignac A, Florin A. Long term posttraumatic growth after breast cancer: prevalence, predictors and relationships with psychological health. *J Clin Psychol Med Settings* 2010;17(1):14-22. doi: 10.1007/s10880-009-9183-6 [published Online First: 2010/01/19]

53. Cadell S, Suarez E, Hemsworth D. Reliability and validity of a French version of the posttraumatic growth inventory. *Open Journal of Medical Psychology* 2015;4:53-65. doi: 10.4236/ojmp.2015.42006

54. Maercker A, Langner R. Persönliche Reifung (personal growth) durch Belastungen und Traumata: Validierung zweier deutschsprachiger Fragebogenversionen. [Posttraumatic personal growth: Validation of German versions of 2 questionnaires.]. *Diagnostica* 2001;47(3):153-62. doi: 10.1026/0012-1924.47.3.153

55. Campbell-Sills L, Stein MB. Psychometric analysis and refinement of the Connor-davidson Resilience Scale (CD-RISC): Validation of a 10-item measure of resilience. *J Trauma Stress* 2007;20(6):1019-28. doi: 10.1002/jts.20271 [published Online First: 2007/12/25]

56. Sarubin N, Gutt D, Giegling I, et al. Erste Analyse der psychometrischen Eigenschaften und Struktur der deutschsprachigen 10- und 25-Item Version der Connor-Davidson Resilience Scale (CD-RISC). [First analysis of the 10- and 25-item German version of the Connor-Davidson Resilience Scale (CD-RISC) regarding psychometric properties and components.]. *Zeitschrift für Gesundheitspsychologie* 2015;23(3):112-22. doi: 10.1026/0943-8149/a000142

57. Zimet GD, Powell SS, Farley GK, et al. Psychometric characteristics of the Multidimensional Scale of Perceived Social Support. *J Pers Assess* 1990;55(3-4):610-7. doi: 10.1080/00223891.1990.9674095 [published Online First: 1990/01/01]

For peer review only - http://bmjopen.bmj.com/site/about/guidelines.xhtml

58. Denis A, Callahan S, Bouvard M. Evaluation of the French version of the multidimensional scale of perceived social support during the postpartum period. *Matern Child Health J* 2015;19(6):1245-51. doi: 10.1007/s10995-014-1630-9 [published Online First: 2014/11/05]

59. Studer J, Baggio S, Dupuis M, et al. Substance use in young Swiss men: The interplay of perceived social support and dispositional characteristics. *Substance Use & Misuse* 2017;52(6):798-810. doi: 10.1080/10826084.2016.1264966

60. Pocnet C, Antonietti J-P, Strippoli M-PF, et al. Individuals' quality of life linked to major life events, perceived social support, and personality traits. *Quality of Life Research* 2016;25(11):2897-908. doi: 10.1007/s11136-016-1296-4

61. Kristensen TS, Hannerz H, Høgh A, et al. The Copenhagen Psychosocial Questionnaire--a tool for the assessment and improvement of the psychosocial work environment. *Scand J Work Environ Health* 2005;31(6):438-49. doi: 10.5271/sjweh.948 [published Online First: 2006/01/24]

62. Dupret É, Bocéréan C, Teherani M, et al. Le COPSOQ : un nouveau questionnaire français d'évaluation des risques psychosociaux. *Santé Publique* 2012;24(3):189-207. doi: 10.3917/spub.123.0189

63. Nübling M, Stößel U, Hasselhorn HM, et al. Measuring psychological stress and strain at work - Evaluation of the COPSOQ Questionnaire in Germany. *Psychosoc Med* 2006;3:Doc05. [published Online First: 2006/01/01]

64. Postmes T, Haslam SA, Jans L. A single-item measure of social identification: reliability, validity, and utility. *Br J Soc Psychol* 2013;52(4):597-617. doi: 10.1111/bjso.12006 [published Online First: 2012/11/06]

65. Aiken LH, Sermeus W, Van den Heede K, et al. Patient safety, satisfaction, and quality of hospital care: cross sectional surveys of nurses and patients in 12 countries in Europe and the United States. *British Medical Journal* 2012;344:1-14. doi: 10.1136/bmj.e1717

66. Pelz C, Schmitt A, Meis M. Knowledge Mapping als Methode zur Auswertung und Ergebnispräsentation von Fokusgruppen in der Markt-und Evaluationsforschung. *Forum Qualitative Sozialforschung / Forum: Qualitative Social Research* 2004; 5(2). https://nbn-resolving.org/urn:nbn:de:0114-fqs0402351.

67. Burgess-Allen J, Owen-Smith V. Using mind mapping techniques for rapid qualitative data analysis in public participation processes. *Health Expect* 2010;13(4):406-15. doi: 10.1111/j.1369-7625.2010.00594.x [published Online First: 2010/06/17]

68. Trochim W, Kane M. Concept mapping: an introduction to structured conceptualization in health care. *Int J Qual Health Care* 2005;17(3):187-91. doi: 10.1093/intqhc/mzi038 [published Online First: 2005/05/06]

69. Vail EF. Knowledge Mapping: Getting Started with Knowledge Management. *Information Systems Management* 1999;16(4):16-23. doi: 10.1201/1078/43189.16.4.19990901/31199.3

Figure legends:

1. Figure 1: Study's theoretical framework

2. Summary study timeline (March 2021 to September 2023)

For peer review only - http://bmjopen.bmj.com/site/about/guidelines.xhtml

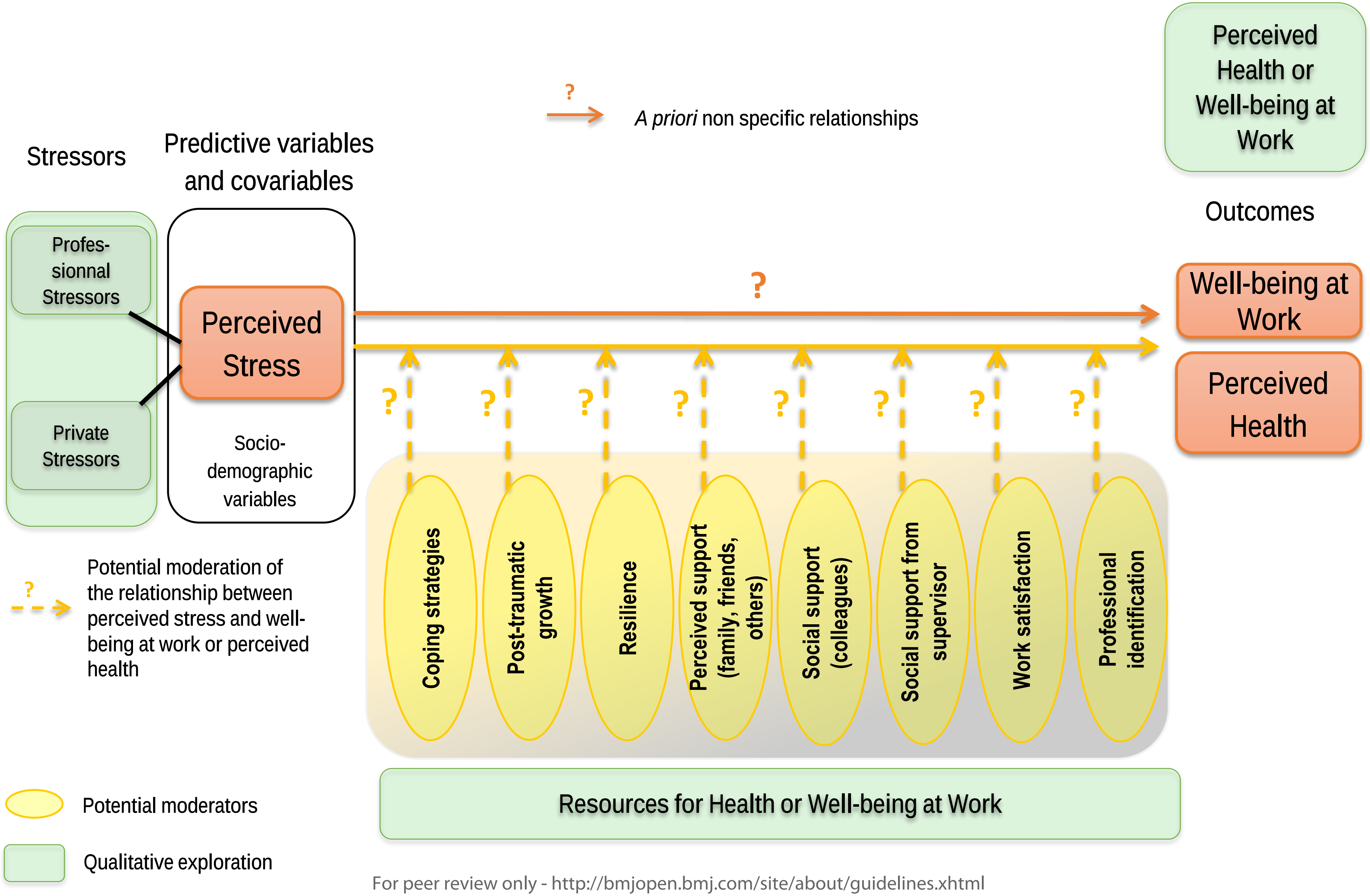

Figure 1: Study's theoretical framework

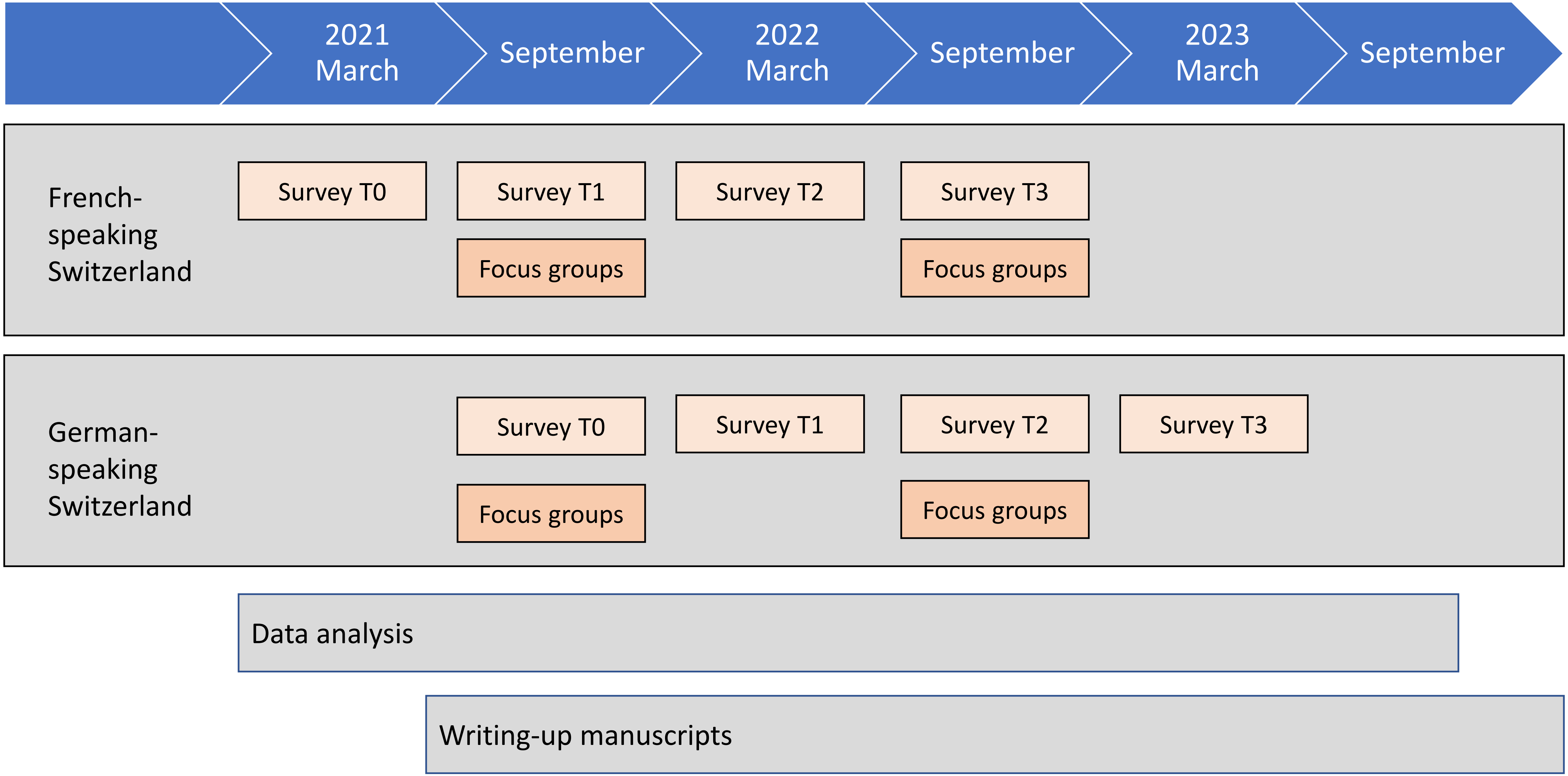

Figure 2: Summary study timeline (March 2021 to September 2023)

BMJ Open

Stressors, self-reported overall health, potential protective factors and the workplace well-being of nurses during the COVID-19 pandemic in Switzerland: a longitudinal mixed-methods study protocol

Journal:	*BMJ Open*
Manuscript ID	bmjopen-2021-057021.R1
Article Type:	Protocol
Date Submitted by the Author:	13-Nov-2021
Complete List of Authors:	Ortoleva Bucher, Claudia; University of Applied Sciences Western Switzerland La Source School of Nursing<br>Delmas, Philippe ; University of Applied Sciences Western Switzerland La Source School of Nursing<br>Oulevey Bachmann, Annie; University of Applied Sciences Western Switzerland La Source School of Nursing<br>Gilles, Ingrid; Center for Primary Care and Public Health, Epidemiology and Health Systems
**Primary Subject Heading**:	Nursing
Secondary Subject Heading:	Public health, Mental health
Keywords:	PUBLIC HEALTH, COVID-19, MENTAL HEALTH

SCHOLARONE™
Manuscripts

TITLE PAGE

a) All authors' full names: Claudia ORTOLEVA BUCHER, Philippe DELMAS, Annie OULEVEY BACHMANN, Ingrid GILLES

b) Corresponding author: Claudia Ortoleva Bucher, HES-SO University of Applied Sciences and Arts Western Switzerland, La Source School of Nursing Sciences, Avenue Vinet 30, 1004 Lausanne, Switzerland. ORCID ID: 0000-0002-8411-4181, E-mail: c.ortolevabucher@ecolelasource.ch, Phone: +41 21 556 40 42

c) Claudia ORTOLEVA BUCHER: La Source School of Nursing Sciences, HES-SO University of Applied Sciences and Arts Western Switzerland, Switzerland

Philippe DELMAS: La Source School of Nursing Sciences, HES-SO University of Applied Sciences and Arts Western Switzerland, Switzerland. ORCID ID: 0000-0001-8169-470X

Annie OULEVEY BACHMANN: La Source School of Nursing Sciences, HES-SO University of Applied Sciences and Arts Western Switzerland, Switzerland. ORCID ID: 0000-0001-9537-3587

Ingrid GILLES: Center for Primary Care and Public Health (Unisanté), University of Lausanne, Lausanne, Switzerland. ORCID ID: 0000-0003-2051-4749

d) Keywords: public health, covid-19, mental health, nurses, nursing, health ressources

e) No. of words: 4125

Title: Stressors, self-reported overall health, potential protective factors and the workplace well-being of nurses during the COVID-19 pandemic in Switzerland: a longitudinal mixed-methods study protocol

Abstract

Introduction: The COVID-19 pandemic was making a huge impact on Europe's healthcare systems in the spring of 2020, and most predictive models concurred that pandemic waves were in the offing. Most studies adopted a pathogenic approach to the subject; few used a salutogenic approach. These showed, however, that nurses can retain their health despite a pandemic by mobilising generalised resistance resources'. Our study aims to understand how nurses working in Switzerland's hospitals protected their health and workplace well-being during the COVID-19 pandemic by investigating the moderating effects of the health resources they mobilised against the stressors inherent to the situation. The study aims to explore and describe stressors and the resources nurses used to remain healthy during the COVID-19 pandemic.

Method and Analysis: We will use a concurrent mixed-methods panel design with qualitative analyses ancillary to quantitative analyses. Quantitative data will be collected using electronic questionnaires at four time points over two years. Qualitative data will be collected using focus groups. Nurses from Switzerland's two main linguistic regions who had direct, indirect or no contact with COVID-19 patients will be invited to participate. The *a priori* sample size will be at least 3,631 participants at T0 and 1,852 at T4. Longitudinal structural equation modelling and Knowledge Mapping will be used to analyse quantitative and qualitative data, respectively. The results derived from the two data types will then be compared and discussed using a side-by-side approach to determine whether they agree or disagree and how they complement each other to achieve our aims.

Ethics and Dissemination: Nurses will receive an electronic informed consent form. The data collected will be stored on a secure server at the authors' institution. This research project was approved by the Human Research Ethics Committee of the Canton of Vaud (2020-02845).

For peer review only - http://bmjopen.bmj.com/site/about/guidelines.xhtml

STRENGTHS AND LIMITATIONS OF THIS STUDY

- The use of a salutogenic approach focussing on nurses' resources or protective health factors for dealing with stressful situations.
- The use of a mixed-methods design to enrich the examination of possible links between nurses' resources or protective health factors and workplace well-being (the study's key contribution).
- The use of a longitudinal design to examine changes in our two outcomes over time (quality of life and workplace well-being), in exposure to COVID-related stressors, in factors protective of health and in associations between them.
- The potential design adaptations needed due to uncertainties about the ongoing pandemic situation.
- The risks of attrition due to lengthy data collection.

For peer review only - http://bmjopen.bmj.com/site/about/guidelines.xhtml

INTRODUCTION

The COVID-19 pandemic began in China in December 2019. Despite the imposition of drastic confinement measures, Switzerland's healthcare system was very badly affected—in some countries, they were completely overwhelmed [1 2].

Pandemic situations expose nurses to different types of stressors, such as performing unusual tasks in unusual settings (e.g., units specially dedicated to the care of infected patients) and adapting to unusual work shifts. Such exposure has been associated with high levels of psychological distress [3-6] and headaches [7]. During the SARS epidemic, studies reported that between 17.3% and 75.3% of healthcare workers (HCWs) presented with mental health problems [8-12]. Two recent systematic reviews regarding SARS, together with the current literature on COVID-19, have shown that HCWs who worked in COVID-19 units were at a high risk of developing the same psychological symptoms [3 6]. Compared with other HCWs, nurses reported higher stress levels, more psychopathological symptoms and more post-traumatic stress symptoms [13 14]. These problems can persist for up to two years after the end of an epidemic [15]. Such stressful situations generally result in greater cynicism vis-à-vis patients, more mistakes and safety issues, poorer communication among nurses and increased costs for healthcare services in terms of both human and financial resources [16 17]. Moreover, nurses are at a higher risk of burnout [17-19]. Most studies in this area have taken a pathogenic approach, focussing on identifying diseases, symptoms and risk factors [3 4]. Few studies have adopted a salutogenic approach focussing on well-being and identifying the health resources mobilised by HCWs to help them cope with their contextual stressors. Salutogenesis is "a scholarly orientation focusing attention on the study of the origins of health and assets for health, contra the origins of disease and risk factors" [20]. It considers health as a continuum from optimal health to disease. When developing preventive actions, and instead of simply considering risk factors, salutogenesis also aims to highlight the so-called 'factors protective of health' or the 'generalised health resources' that actively support health [20]. Studies adopting this approach have shown that nurses can retain their health in such situations by mobilising 'generalised resistance resources' [20]. For example, two preliminary studies conducted in Belgium[21] and Switzerland's French-speaking region [22] have shown that the pandemic's impact has not been entirely negative and that it had a positive impact on nurses at both the personal and professional levels. In Belgium, Lecocq et al. [23] conducted a qualitative study with 100 nurses working in different sectors of activity (units, medical, psychiatric units) of University Hospital of Brussels between March and June 2020. They found core themes that structured the experience of professionals during this unprecedented period. Actually, if professionals expressed "fear and lack of safety" concerning the uncertainty of this period, they also highlighted positive aspects such as the fact to being able to engage in "authentic relationship with patients", functioning in real support teams with less

For peer review only - http://bmjopen.bmj.com/site/about/guidelines.xhtml

supervision from the hierarchy and "drawing on one's resources to stay healthy". It turns out that this period of pandemic has also afforded nurses the opportunity to highlight the added value that they bring to care and raise importance of their role in the eyes of the population.

Thus, nurses managed to find the resources to meet the challenges they faced. Gaining clearer insight into how nurses maintained their health in the face of a pandemic is essential. The salutogenic perspective has rarely been explored in a pandemic context despite its real-world applications and may provide blueprints for developing preventive interventions aimed at maintaining HCWs' health and workplace well-being during times of pandemic. Therefore, this research project will adopt a salutogenic perspective.

Most studies conducted on this topic to date have been atheoretical [3 4]. Accordingly, the relationships between concepts have varied widely. Depression, for example, has been considered a predictor and an outcome [3 4]. To describe, explain and understand the nature and meaning of phenomena, and to develop interventions in the health field, research should be based on established models [24]. Our theoretical framework will be the Neuman Systems Model (NSM), considered a salutogenic model according to the criteria of both Lindström and Eriksson [25] and Mittelmark and Bauer [20]. The NSM considers health to be in a dynamic equilibrium in which human beings can retain, attain and retain stability, and in which perceived wellness is viewed as a manifestation of health [26]. Stressors are considered neutral *a priori*; their impact depends on circumstances, the moment, and the individual's perception of them and ability to cope with them ('eustress' and 'stress') [26]. An individual's ability to retain health and well-being is subject to two mechanisms: reducing exposure to stressors and activating factors protective of health. These protective factors may be physiological, psychological, socio-cultural, developmental or spiritual, and they may act as moderators between stressors and health [26 27]. Finally, an individual's ability to retain their health is accordion-like: when strengthened by primary preventive interventions, it can develop and become more effective. The NSM's theoretical framework seems well suited for our study because it enables an exploration of: 1) the stressors to which HCWs' health is exposed; 2) their relationships with HCWs' overall health and well-being over time; and 3) the mechanisms used to stay healthy despite this exposure (Figure 1).

Another limitation identified in studies exploring the impact of the COVID-19 pandemic or other epidemics (SARS or MERS) on HCWs' health is their cross-sectional design and/or post-pandemic research design [3 4]. These allowed for no examination of changes in nurses' health over time during the pandemic episode, nor for any determination of causality between exposure and outcomes. Longitudinal studies are needed to establish causal relationships between stressors, moderators and outcomes in the context of COVID-19 [28]. To the best of our knowledge, there are only three longitudinal

For peer review only - http://bmjopen.bmj.com/site/about/guidelines.xhtml

studies analyzing nurses' health during COVID-19 [29-31]. In Switzerland, Fuchs *et al.*, [30] conducted a longitudinal mixed-methods study, which started in April and was planned to end in December 2020, to analyse HCWs' mental health using a pathogenic perspective (outcomes include symptoms such as anxiety, depression and PTSD). In Canada, Richardson *et al*. have been monitoring HCWs for 18 months to measure their well-being and levels of distress [29]. In Portugal, Pinho et al. have been monitoring use of mental health promotion strategies by nurses is important to reduce stress, anxiety, and depression symptoms. Our proposed longitudinal mixed-methods study using a salutogenic approach would complement these three timely studies.

Because this unprecedented pandemic situation is ongoing, with no end in sight [28], our research project based on the NSM has the following objectives:

- i) to understand how nurses working in Switzerland's hospitals retained their health and workplace well-being throughout the COVID-19 pandemic (mixed-methods);
- ii) to explore the stressors experienced by nurses throughout the months of the pandemic (qualitative methods);
- iii) to measure changes in their experience of these stressors and in any possible repercussions on their overall health and workplace well-being during the pandemic (quantitative methods);
- iv) to explore and describe the resources used to remain healthy during the pandemic (quantitative and qualitative methods).

METHODS AND ANALYSIS

Study design overview

We will use a concurrent mixed-methods panel design. Quantitative analyses [32] will use data from four successive waves of self-reporting questionnaires at six-month intervals (T0,; T1, at six months; T2, at 12 months; and T3 at 18 months). In our study, T0 refers to the study baseline and not the time before the beginning of the pandemic. We are conscious that the situation was critical for health care workers already a year before the beginning of our study and that the study begins at a time when the resistance of the professionals has already been put to the test with a level of stress that is probably higher than usual. Actually, the preparation of the study the construction of the study began at the end of the first wave of COVID-19, i.e., in the summer of 2020, but the important waves of the autumn pushed us to plan a beginning of the study in the beginning of 2021 in order not to solicit the professionals more at a critical period. What will be important to observe in our study will be the

For peer review only - http://bmjopen.bmj.com/site/about/guidelines.xhtml

evolution over time of this extraordinarily extensive situation, keeping in mind that we will not have information on the initial level of stress among nurses. This survey started in March 2021 in French-speaking Switzerland and will start in September 2021 in German-speaking Switzerland (Figure 2). Qualitative interviews will take place in parallel using two focus group (FG) phases (in September 2021 and 2022) in the respective linguistic regions.

Both methodological parts are detailed below, and several reasons led to their selection (following the STROBE guidelines). Concerning the mixed-methods approach, it is recommended that multi-faceted research questions use more than one method of investigation [33], and, in our case, understanding stressors requires just such in-depth exploration. A longitudinal design will be necessary to evaluate changes over time [34]. According to Wang *et al*. [34], at least three time points are necessary to provide a robust picture of changes in socio-psychological variables and how they relate to one another. Based on the most common longitudinal wave time-point intervals reported in the literature (6–12-months) (2) and our own research experience (4, 5), we chose a 6-month interval.

Quantitative part

A self-administered electronic survey covering socio-demographic and psychological questionnaire items will be sent to nurses at 15 hospitals and clinics in the French- and German-speaking regions of Switzerland by email. No sampling strategy will be applied; every eligible nurse will be contacted. A communication strategy to encourage participation has been developed (emails announcing the study, reminders). The data collection phase at each time point (T0 to T3) will last one month.

Population and sampling

The population includes all the nurses working in our partnering healthcare institutions at baseline, regardless of whether they have had direct, indirect or no contact with patients infected with COVID-19. Fifteen institutions have already agreed to facilitate the study, making our potential population 5,187 nurses working in different hospitals in Switzerland's German- and French-speaking regions.

Inclusion criteria at baseline are: (i) having a long-term (not temporary) contract at the institution selected; (ii) working at least 50% of a full-time equivalent position; (iii) being able to read and understand French or German; and (iv) having signed the written informed consent form. Exclusion criteria at baseline are: (i) being a nurse manager; (ii) not working at the institution during the COVID-19 crisis; and (iii) being a student nurse during the COVID-19 crisis.

For peer review only - http://bmjopen.bmj.com/site/about/guidelines.xhtml

Recruitment targets

We used previous relevant studies to estimate participation and attrition rates. A recent Swiss survey of HCWs' overall health had a response rate of 37% [22]. Concerning attrition rates, Niu *et al*. [35] expect 20% in their study of nurse sleep quality and Kovner *et al*. [36] reported a 30% attrition rate in their panel study of nurse turnover. The Swiss Household Panel survey recorded attrition rates from 13% to 18% [37]. We therefore estimate a 20% attrition rate at each data collection time point. Thus, we expect our accessible population of 5,187 nurses at T0 to fall to 3,631 at T1 (80% of T0 staff), 2,905 at T2 (80% of T1 staff), 2,324 at T3 (64% of T1 staff), with a final sample of 1,852 at T4 (51% of T1 staff).

Measurement

The questionnaire is composed of the following scales:

Predicting variable

The Perceived Stress Scale (PSS) [38], in validated French (Cronbach's α=.78–.87) and German versions (Cronbach's α: .84) [39 40], is a 10-item scale to evaluate the degree to which life situations are generally perceived as threatening, that is, as unpredictable, uncontrollable and painful [41].

Outcome variables

The Psychological Well-Being Scale, developed by Diener *et al*. [42] and adapted for the workplace by Fisher [43], in validated French and German versions (Cronbach's α=.88 and confirmed the scale's one-dimensional structure) [44-46], is an 8-item scale to evaluate workplace well-being and measures self-perceived functioning in areas such as self-esteem, purpose and relationships. Convergent validity analyses have shown good correlations (.43>r< .73) with the Basic Needs Satisfaction Scale[47], Ryff's Psychological Wellbeing Scale[48], the Satisfaction with Life Scale[49] , and the Life Orientation Test-Revise[44].

The World Health Organization Quality of Life – BREF (WHOQOL-BREF) [50], in validated French and German versions [51 52], is a 26-item short version of the 100-item WHOQOL scale covering four domains—physical health, mental well-being, social relations and work environment—that measure self-perceived quality of life. Psychometric properties have proved good to excellent for the original version[50], and the French and German versions showed Cronbach's α>.65 for all dimensions[51 52].

Mediating variables

The Brief-COPE [53], in validated French and German versions [54 55], is a 28-item scale evaluating several coping strategies. The French and German subscales have obtained Cronbach's α of .50–.90 (total score is not representative).

For peer review only - http://bmjopen.bmj.com/site/about/guidelines.xhtml

Moderating variables

The Post-traumatic Growth Inventory-Short Form (PTGI-SF) [56], in validated French and German versions [57-59], is a 10-item inventory evaluating post-traumatic growth, that is, positive psychological change experienced following a traumatic event. The PTGI has demonstrated good internal coherence (Cronbach's α: French version: .90 for total score) and acceptable test-retest (r=.71).

The 10-item Connor–Davidson Resilience Scale (CD-RISC®) [60], in validated French and German versions [60 61], measures a person's ability to bounce back when confronted with the difficulties that may arise in life. This 10-item unidimensional version possesses excellent psychometric properties, better than those of the original 25-item version, and its use is suited to large-scale epidemiologic studies [60 62 63]. Its 3-month test-retest is good [64].

The Multidimensional Scale of Perceived Social Support (MSPSS) [65], in validated French and German versions [66-68], is a 12-item scale measuring perceived social support from family, friends and significant others. Cronbach's α range from .91 to .94 for the French and German version [66-68]. Its 4-month test-retest has ranged from .72 to .85 [65].

The Copenhagen Psychosocial Questionnaire (COPSOQ) [69], in validated French and German versions [70 71], is a comprehensive tool for assessing psychosocial risk in the workplace through 24 core dimensions covering four aspects of work: work environment, health, well-being and personality. We will use three of these dimensions: dimensions of social support from colleagues, dimensions of social support from supervisors and dimensions of satisfaction with job quality. The COPSOQ has been used in various work contexts in different languages, including French [70] and German [71] and has been constantly improved, the latest version having been proposed by its authors in 2018 [72]. These dimensions have good internal consistency (all α>.80).

Professional identification will be measured using the single-item measure of social identification proposed by Postmes *et al*. [73], which can be adapted to any social group, including professional ones. Its simple wording ("I identify with [target group]" reduces interpretation bias and translation issues; its 7-point rating scale ranges from 1 = fully disagree to 7 = fully agree. The tool has been tested against more classic scales such as the 5-item Social Identity Scale [74] and the 14-item Leach Scale [75] and showed good convergent, divergent and test-retest validity. Moreover, a meta-analysis found this single-item measure to possess stronger reliability compared with other "tried-and-trusted" single-item measures [73].

Socio-demographic variables. Information regarding gender, age, family situation, and number of children, employment rate, years since last diploma, years of experience in current department, level of exposure to COVID-19 (nurses working in COVID-19 care units; nurses working in institutions

For peer review only - http://bmjopen.bmj.com/site/about/guidelines.xhtml

that admitted infected patients, but not in COVID-19 units; nurses working in institutions that did not admit infected patients) and continuing professional development program completed will be collected. As suggested by Aiken *et al*. [76], hospitals' organisational indicators (e.g. nurse–patient ratios, work schedules, average time spent with patients per shift) will be collected at each measurement point.

Quantitative data analyses

Standard data-quality checks will be performed to remove unreliable values from the database, and descriptive statistics will be computed to qualify those data.

As specified in the introduction section, the Neuman Systems Model (NSM) is used as conceptual framework in this study. According to this model, the ability of individuals to maintain their health and well-being against a-priori neutral stressors is conditioned on the mobilization of protective factors. It has thus been proposed that some factors could moderate the negative association between stressors and the normal line of defence what would result in an optimal health. However, the mechanisms underlying the action of protective factors as well as the identification of these factors are still discussed[27]. Concerning the identification of factors, the underlying postulate of the NSM is that they vary according to the situation. That is why we selected relevant variables on the basis of the existing literature on previous outbreaks, but also on the basis of studies using a salutogenic approach specifically in the COVID-19 context. For example, resilience, social support (including this provided by colleagues and supervisors), post-traumatic growth and identification to professional group appeared to be key resources for nurses during critical situations [15 77-85]. The aim of quantitative analyses will be thus to identify, in a longitudinal perspective, which factors were of most importance during the pandemic to buffer the effect of stressors faced by nurses on their health and professional well-being (our outcomes). To reach these objectives, association between perceived stress and well-being will subsequently be modelled using mixed-effects regressions. Random effects are necessary to model the data's longitudinal structure. Linear models will be the default choice, but we will not rule out resorting to data manipulation (e.g. dichotomisation) or alternative models (e.g. logistic models) if the data's structure requires it. The moderating effects of post-traumatic growth, resilience, perceived support, coping strategies, job satisfaction and professional identity will be evaluated by entering the estimated interactions between these variables and perceived stress into the regression models. A frequentist probability approach will be used for all our analyses. All calculations will be run on the latest available stable version of R software. Statistical significance will be set at p-value < .05.

Qualitative part

For peer review only - http://bmjopen.bmj.com/site/about/guidelines.xhtml

We will use a knowledge mapping approach [86]. Mind mapping is a useful graphical format for representing key themes raised during FGs. It can help stimulate and galvanise discussions and keep them on track, enhance transparency and group ownership of the data analysis process, and enable a rapid dynamic to develop between data collection and feedback [87]. This pragmatic approach is considerably faster than traditional methods used for analysing FGs, but it produces broadly similar results [87]. Participants are actively involved in interpreting the resulting mind maps [88]. The advantages of mapping include its 'free form' and unconstrained structure, meaning there are no limits to the ideas and links that can be made and no need to retain an ideal structure or format. Mapping thus promotes creative thinking and encourages brainstorming. The three steps to structuring a knowledge mapping approach are: (1) developing interview guidelines, (2) conducting FG interviews and (3) analysis and conclusion [86].

Population

The target population will consist exclusively of nurses who respond to the questionnaire at T0 and indicate their interest in participating in the study's future stages by giving their written informed consent at that time. The interested nurses in each linguistic region will be divided into three groups according to their degree of contact with COVID-19 patients: 1) nurses in direct contact with infected patients (worked in COVID-19 care units); 2) nurses in indirect contact because their institution admitted infected patients, but they did not work in COVID-19 care units; 3) nurses with no contact because their institutions did not admit infected patients.

Sampling and recruitment targets

The research team will select three samples of nurses to participate in FGs in each of the two linguistic regions 12 months apart. Nurses will be allowed to participate in the FGs during their paid work hours. If there are too many volunteers, participants will be selected using a convenience sampling method based on socio-demographic characteristics such as age, their institution's geographical location, whether their COVID-19 experience had been on their usual ward, and to foster heterogeneity among the FG participants. If there has been attrition in the FGs at T3, groups will be completed with new participants selected in the same way.

Data collection

Investigators began by developing a semi-structured interview guide based on the NSM and tested it on two nurses not participating in the study. Several pathways will be explored: lived experiences and

stressors, health, well-being at work, as well as the evolution of these components over time and the resources mobilised to retain them.

FG discussion will last about 1.5 hours and will be held by videoconference that facilitates mutual trust and ensure confidentiality. Participants will be formally asked for their consent before conversations are recorded. A moderator (a senior researcher) will lead each FG, assisted by a scientific collaborator. Both will be trained beforehand and will receive precise guidelines to ensure standardised procedures. All the topics in the interview guide will be discussed.

A knowledge map of each topic discussed will be drawn on a flip chart, resulting in a synthesis formed by a complex network of representations in text: “A knowledge map is the visual display of captured information and relationships […]” [89]. Central concepts within each topic will be arranged in relation to one another, and recurring concepts will be highlighted. Before moving from one topic to the next, the moderator will ensure that every input has been documented and that participants validate the map to ensure that the researchers have properly understood each point as they have been expressed [86]. The knowledge maps will also be considered as protocols for the FG discussions.

Qualitative data analysis

Qualitative analysis will begin during the FG sessions when participant feedback confirms that each map fully summarises each topic’s discussion. Knowledge maps will then be digitised and merged by interview topic. They will then be translated into English to enable the complete integration of the different German and French language maps. The integrated maps in English will then be discussed, and the qualitative research team will cluster themes. Each cluster will be described with regards to the study’s aims.

To ensure rigour in the qualitative data collection process, the investigators will maintain a logbook of methodological, theoretical and personal notes.

Integrating quantitative and qualitative data

This research is based on a concurrent mixed design (QUANTI/quali). At T1 and T3 qualitative and quantitative data will be collected in parallel. The qualitative data are ancillary to the quantitative data. The interview guide for collecting the former will therefore not be dependent on the results of the latter.

The qualitative data will shed light on the experience of the crisis at T1 and T3, its links with perceived health and well-being at work overtime, and the QUANTI health maintenance measures will highlight the nurses' use of protective factors/health resources during the pandemics

The integration of the data (QUANTI/quali) will be done through a process of comparison on two occasions (T1 and T3). For this purpose, we plan to bring together the QUANTI and Quali research teams from both language regions in workshops. Similarities and differences between QUANTI and Quali results will be investigated and discussed. The mixed product will consist of interpretations or decisions developed in the research team that take into account the explicit interdependencies between QUANTI and Quali data [32]

Patient and public involvement

Patients and/or the public were involved in the design, conduct, reporting or dissemination plans of this research. Refer to the Methods and analysis section for further details.

Study status

Participant recruitment for the survey's first round (T0) began in March 2021 in French-speaking Switzerland. Subsequent rounds are planned for September 2021 (T1), March 2022 (T2) and September 2022 (T3). Participation in German-speaking Switzerland will occur with a 6-month delay: T0 in September 2021, T1 in March 2022, T2 in September 2022 and T3 in March 2023. This time lag will be used to collect qualitative data. Thus, the FGs for French- and German-speaking Switzerland are scheduled for September 2021 and September 2022, respectively.

Ethics and dissemination

This project was approved by the Human Research Ethics Committee of the Canton of Vaud (project no. 2020-02845). Each partner institution will designate a reference person. The research team will send that person an anonymous link to the online questionnaire and an information letter and a standard study invitation email to be adapted for each institution.

At T0, each reference person will send out their adapted information email to their institution's potential participants (specifying that the study is longitudinal and includes four waves). These nurses will be free to accept or decline participation. At the end of the questionnaire, participants who wish to join in the next waves and FGs will be asked to provide an email address so the research team can contact them directly.

Participants will be able to withdraw from the study at any time. Email addresses will be kept by the research team member responsible for the logistical aspects of data collection (sending invitation emails and reminders). Other research team members will not have access to this information. Nominative information (e.g. emails) will be deleted at the end of the study.

For peer review only - http://bmjopen.bmj.com/site/about/guidelines.xhtml

Participation in both the quantitative and qualitative parts of this study will be voluntary—nurses will be under no obligation. Electronic 'clicked' consent will be collected from all participants in the quantitative part on a form stating that their data may be used and shared with other researchers in an anonymised form and that they will not be identified nominatively in any outputs. All participants will have a unique anonymous identifier, and only this identifier will appear in the datasets. Separate files with personal details will be kept securely, will not be shared with other researchers and will be deleted on completion of the project. At least 72 hours before collecting qualitative data in FGs, participants will receive an information letter and a consent form for signature. Recordings will not be shared, and transcriptions will be anonymised. Personal data enabling the identification of participants will be kept separate from survey responses and will be deleted when data collection is complete. Any data to be made public will be entirely anonymous.

Nurses will be under no obligation to answer any or all of the questions, and they will be free to interrupt questionnaires at any time. In this event, answers already provided will not be registered.

Results will be disseminated in several ways. The first persons to benefit from this research will be the participants themselves. A document containing the study's highlights will be prepared specifically for them and made available on our partner institutions' websites or on demand. These institutions will hold conferences open to the public. Scientific publications will be addressed to the French-, English- and German-speaking scientific communities (e.g. *BMC Health and Services Research, Journal of Clinical Nursing, International Journal of Nursing Research, Recherche en Soins Infirmiers, etc.*). Results will also be disseminated at international and local conferences. Finally, a research report containing the study's key findings will be produced for the libraries of the different partnering institutions and for the funding partner.

Study significance

We believe that this study will make a notable contribution to advancing scientific knowledge on HCWs' health and workplace well-being during pandemics, particularly on the following aspects: a) analysing changes in the effects of various potential factors protective of nursing health and workplace well-being; b) developing knowledge using an established theoretical framework in a novel context; and c) guiding the development of new preventive interventions to support nurses in times of pandemic. Several elements are worthy of note regarding the potential benefits of this study for our institutional field partners. Study findings could be integrated into quality improvement processes at participating institutions and could inform the development of support programmes there for preventing disease and promoting health among their HCWs.

For peer review only - http://bmjopen.bmj.com/site/about/guidelines.xhtml

Embedding the NSM's theoretical framework in a salutogenic approach [26] will provide us with an essential insight into what protects nurses' overall health and workplace well-being during a pandemic such as the one we are living through. It will shed light on the different stressors that they are exposed to. This is a vital step towards developing preventive interventions geared both to mobilising factors protective of health and to reducing exposure to risk factors. Such interventions would support nurses' overall health and workplace well-being.

Broader impacts

Several elements are worthy of note regarding the potential benefits of this study for our institutional partners. The findings could be integrated into their quality improvement processes and could inform the development of support programmes for preventing disease and promoting health among their staff, particularly nurses.

The results of this research could also be integrated into undergraduate nursing curricula or even continuing professional development programmes at partnering institutions or in healthcare management education programmes in Switzerland. More broadly, they will also support the healthcare system's performance when faced with crises of a similar type.

Author contributions

COB is the principal investigator. COB, AOB, PD and IG contributed to the study design. IG hosted the survey. COB, PD and IG are mainly collecting the quantitative data and COB, AOB, PD are mainly collecting the qualitative data. COB, AOB, PD and IG are responsible for the data analyses. COB, AOB, PD and IG significantly contributed to the writing of the manuscript. The manuscript was reviewed and edited prior to submission, and all authors agreed on the final version.

Competing interests (for all authors):

The authors have no conflict of interest to declare.

Funding

The study is supported by the Swiss National Science Fundation (10001C_201137/1).

References

1. Remuzzi A, Remuzzi G. COVID-19 and Italy: what next? *The Lancet* 2020;395(10231):1225-28. doi: 10.1016/S0140-6736(20)30627-9

2. Italian Society of Anesthesia RaICS. Clinical ethics recommendations for the allocation of intensive care treatment, in exceptional, resource-limited circumstances 2020 [Available from:

For peer review only - http://bmjopen.bmj.com/site/about/guidelines.xhtml

http://www.siaarti.it/SiteAssets/ News/COVID19%20-%20documenti%20SIAARTI/SIAARTI%20-% 20Covid-19%20-%20Clinical%20Ethics%20Reccomendations.pdf accessed 02.09.2020.

3. Preti E, Di Mattei V, Perego G, et al. The psychological impact of epidemic and pandemic outbreaks on healthcare workers: rapid review of the evidence. *Current Psychiatry Reports* 2020;22(8):43. doi: 10.1007/s11920-020-01166-z

4. Stuijfzand S, Deforges C, Sandoz V, et al. Psychological impact of an epidemic/pandemic on the mental health of healthcare professionals: a rapid review. *BMC Public Health* 2020;20(1):1230. doi: 10.1186/s12889-020-09322-z

5. Pappa S, Ntella V, Giannakas T, et al. Prevalence of depression, anxiety, and insomnia among healthcare workers during the COVID-19 pandemic: A systematic review and meta-analysis. *Brain Behav Immun* 2020;88:901-07. doi: 10.1016/j.bbi.2020.05.026 [published Online First: 2020/05/22]

6. Rajkumar RP. COVID-19 and mental health: A review of the existing literature. *Asian Journal of Psychiatry* 2020;52:102066. doi: https://doi.org/10.1016/j.ajp.2020.102066

7. Chew NWS, Lee GKH, Tan BYQ, et al. A multinational, multicentre study on the psychological outcomes and associated physical symptoms amongst healthcare workers during COVID-19 outbreak. *Brain Behav Immun* 2020;88:559-65. doi: 10.1016/j.bbi.2020.04.049 [published Online First: 2020/04/25]

8. Chong M-Y, Wang W-C, Hsieh W-C, et al. Psychological impact of severe acute respiratory syndrome on health workers in a tertiary hospital. *British Journal of Psychiatry* 2004;185(2):127-33. doi: 10.1192/bjp.185.2.127 [published Online First: 2018/01/02]

9. Goulia P, Mantas C, Dimitroula D, et al. General hospital staff worries, perceived sufficiency of information and associated psychological distress during the A/H1N1 influenza pandemic. *BMC Infectious Diseases* 2010;10(1):322. doi: 10.1186/1471-2334-10-322

10. Lu YC, Shu BC, Chang YY, et al. The mental health of hospital workers dealing with severe acute respiratory syndrome. *Psychotherapy and Psychosomatics* 2006;75(6):370-75. doi: 10.1159/000095443

11. Nickell LA, Crighton EJ, Tracy CS, et al. Psychosocial effects of SARS on hospital staff: survey of a large tertiary care institution. *Canadian Medical Association Journal* 2004;170(5):793. doi: 10.1503/cmaj.1031077

12. Tam CWC, Pang EPF, Lam LCW, et al. Severe acute respiratory syndrome (SARS) in Hong Kong in 2003: stress and psychological impact among frontline healthcare workers. *Psychological Medicine* 2004;34(7):1197-204. doi: 10.1017/S0033291704002247 [published Online First: 2004/10/21]

13. Matsuishi K, Kawazoe A, Imai H, et al. Psychological impact of the pandemic (H1N1) 2009 on general hospital workers in Kobe. *Psychiatry and Clinical Neurosciences* 2012;66(4):353-60. doi: 10.1111/j.1440-1819.2012.02336.x

14. Lai J, Ma S, Wang Y, et al. Factors associated with mental health outcomes among health care workers exposed to Coronavirus Disease 2019. *JAMA Netw Open* 2020;3(3):e203976. doi: 10.1001/jamanetworkopen.2020.3976 [published Online First: 2020/03/24]

15. Maunder RG, Lancee WJ, Balderson KE, et al. Long-term psychological and occupational effects of providing hospital healthcare during SARS outbreak. *Emerging Infectious Disease journal* 2006;12(12):1924. doi: 10.3201/eid1212.060584

For peer review only - http://bmjopen.bmj.com/site/about/guidelines.xhtml

16. Lamiani G, Dordoni P, Argentero P. Value congruence and depressive symptoms among critical care clinicians: The mediating role of moral distress. *Stress Health* 2018;34(1):135-42. doi: 10.1002/smi.2769 [published Online First: 2017/07/01]

17. Austin CL, Saylor R, Finley PJ. Moral distress in physicians and nurses: Impact on professional quality of life and turnover. *Psychol Trauma* 2017;9(4):399-406. doi: 10.1037/tra0000201 [published Online First: 2016/11/01]

18. Christodoulou-Fella M, Middleton N, Papathanassoglou EDE, et al. Exploration of the association between nurses' moral distress and secondary traumatic stress syndrome: Implications for patient safety in mental health services. *Biomed Res Int* 2017;2017:1908712. doi: 10.1155/2017/1908712 [published Online First: 2017/12/07]

19. Papathanassoglou ED, Karanikola MN, Kalafati M, et al. Professional autonomy, collaboration with physicians, and moral distress among European intensive care nurses. *Am J Crit Care* 2012;21(2):e41-52. doi: 10.4037/ajcc2012205 [published Online First: 2012/03/03]

20. Mittelmark M, Bauer G. The Meaning of Salutogenesis. In: Mittelmark M, Sagy S, Eriksson M, et al., eds. The Handbook of Salutogenesis. Cham: Springer 2017.

21. Lecocq D, Delmas P, Antonini M, et al. Comparing feeling of competence regarding humanistic caring in Belgian nurses and nursing students: A comparative cross-sectional study conducted in a French Belgian teaching hospital. *Nursing open* 2020;8(1):104-14. doi: 10.1002/nop2.608

22. Antonini, M. , Perriraz M, Delmas P, et al. Nurses and healthcare assistants' views of their institution's response to first wave of COVID-19 pandemic and their intentions to remain in their current workplace: a first glance at the ProH-COVID project *Bulletin of the Swiss Sociological Association* 2020;157:20-25.

23. Lecocq D, Delmas P, Antonini M, et al. Comparing feeling of competence regarding humanistic caring in Belgian nurses and nursing students: A comparative cross-sectional study conducted in a French Belgian teaching hospital. *Nursing Open* In press doi: 10.1002/nop2.608

24. Fawcett J, Desanto-Madeya S. Contemporary nursing knowledge : analysis and evaluation of nursing models and theories (3rd ed.). . Philadelphia: F. A. Davis Company 2013.

25. Lindström B, Eriksson M. La salutogenèse : petit guide pour promouvoir la santé. Québec: PUL 2012.

26. Neuman B, Fawcett J. The Neuman Systems Model (5th ed.). Boston: Pearson Education 2011.

27. Gigliotti E. New advances in the use of Neuman's Lines of Defense and Resistance in Quantitative Research. *Nurs Sci Q* 2012;25(4):336-40. doi: 10.1177/0894318412457054

28. Flynn R, Riches E, Reid G, et al. Rapid review of the impact of COVID-19 on mental health. Edinburgh: Public Health Scotland, 2020.

29. Richardson D. Moral distress and well-being in health care workers during COVID-19 pandemic: A longitudinal study Canada: Canadian Psychological Association; 2020 [Available from: https://cpa.ca/abstract/moral-distress-and-well-being-of-health-care-workers-during-the-covid-19-pandemic-a-longitudinal-study/ accessed 13.09.2020.

30. Fuchs A, Abegglen S, Berger-Estilita J, et al. Distress and resilience of healthcare professionals during the COVID-19 pandemic (DARVID): study protocol for a mixed-methods research project. *BMJ Open* 2020;10(7):e039832. doi: 10.1136/bmjopen-2020-039832 [published Online First: 2020/08/02]

31. Pinho L, Correia T, Sampaio F, et al. The use of mental health promotion strategies by nurses to reduce anxiety, stress, and depression during the COVID-19 outbreak: A prospective cohort study. *Environmental Research* 2021;195:110828. doi: https://doi.org/10.1016/j.envres.2021.110828

For peer review only - http://bmjopen.bmj.com/site/about/guidelines.xhtml

32. Creswell J, Creswell J. Research design: Qualitative, quantitative, and mixed methods approaches. Los Angeles: Sage Publications 2018.

33. Creswell J, Plano CV. Designing and Conducting Mixed Methods Research, 3rd ed. . Thousand Oaks, CA: Sage Publications Inc. 2013.

34. Wang M, Beal DJ, Chan D, et al. Longitudinal Research: A Panel Discussion on Conceptual Issues, Research Design, and Statistical Techniques. *Work, Aging and Retirement* 2016;3(1):1-24. doi: 10.1093/workar/waw033

35. Niu SF, Miao NF, Liao YM, et al. Sleep quality associated with different work schedules: a longitudinal study of nursing staff. *Biol Res Nurs* 2017;19(4):375-81. doi: 10.1177/1099800417695483 [published Online First: 2017/06/20]

36. Kovner CT, Djukic M, Fatehi FK, et al. Estimating and preventing hospital internal turnover of newly licensed nurses: A panel survey. *Int J Nurs Stud* 2016;60:251-62. doi: 10.1016/j.ijnurstu.2016.05.003 [published Online First: 2016/06/15]

37. Voorpostel M, Tillmann R, Lebert F, et al. Swiss Household Panel Userguide (1999-2016), Wave 18, December 2017. Lausanne: FORS, 2017.

38. Cohen S, Kamarck T, Mermelstein R. A global measure of perceived stress. *J Health Soc Behav* 1983;24(4):385-96. [published Online First: 1983/12/01]

39. Bellinghausen L, Collange J, Botella M, et al. [Factorial validation of the French scale for perceived stress in the workplace]. *Sante Publique* 2009;21(4):365-73. [published Online First: 2010/01/28]

40. Klein EM, Brähler E, Dreier M, et al. The German version of the Perceived Stress Scale - psychometric characteristics in a representative German community sample. *BMC Psychiatry* 2016;16:159. doi: 10.1186/s12888-016-0875-9 [published Online First: 2016/05/25]

41. Quintard B. Du stress objectif au stress perçu. In: Bruchon-Schweitzer M, Dantzer R, eds. Instroduction à la psychologie de la santé. Paris: PUF 1994.

42. Diener E, Wirtz D, Tov W, et al. New well-being measures: short scales to assess flourishing and positive and negative feelings. *Social Indicators Research* 2010;97(2):143-56. doi: 10.1007/s11205-009-9493-y

43. Fisher CD. Conceptualizing and measuring wellbeing at work. *Wellbeing* 2014:1-25. doi: doi:10.1002/9781118539415.wbwell018

10.1002/9781118539415.wbwell018

44. Villieux A, Sovet L, Jung S-C, et al. Psychological flourishing: Validation of the French version of the Flourishing Scale and exploration of its relationships with personality traits. *Personality and Individual Differences* 2016;88:1-5. doi: 10.1016/j.paid.2015.08.027

45. Giuntoli L, Ceccarini F, Sica C, et al. Validation of the Italian versions of the Flourishing Scale and of the Scale of Positive and Negative Experience. *SAGE Open* 2017;7(1):2158244016682293. doi: 10.1177/2158244016682293

46. Esch T, Jose G, Gimpel C, et al. [The Flourishing Scale (FS) by Diener et al. is now available in an authorized German version (FS-D): application in mind-body medicine]. *Forsch Komplementmed* 2013;20(4):267-75. doi: 10.1159/000354414 [published Online First: 2013/09/14]

47. Deci EL, Ryan RM. The "what" and "why" of goal pursuits: Human needs and the self-determination of behavior. *Psychological Inquiry* 2000;11(4):227-68. doi: 10.1207/S15327965PLI1104_01

For peer review only - http://bmjopen.bmj.com/site/about/guidelines.xhtml

48. Ryff CD, Singer BH. Best news yet on the six-factor model of well-being. *Social Science Research* 2006;35(4):1103-19. doi: 10.1016/j.ssresearch.2006.01.002

49. Diener E, Emmons RA, Larsen RJ, et al. The satisfaction with life scale. *Journal of Personality Assessment* 1985;49(1):71-75. doi: 10.1207/s15327752jpa4901_13

50. Skevington SM, Lotfy M, O'Connell KA. The World Health Organization's WHOQOL-BREF quality of life assessment: psychometric properties and results of the international field trial. A report from the WHOQOL group. *Qual Life Res* 2004;13(2):299-310. doi: 10.1023/b:Qure.0000018486.91360.00 [published Online First: 2004/04/17]

51. Leplège A, Debout C. Mesure de la qualité de vie et science des soins infirmiers. *Recherche en soins infirmiers* 2007;88(1):18-24. doi: 10.3917/rsi.088.0018

52. Angermeyer MC, Kilian R, Matschinger H. WHOQOL - 100 und WHOQOL - BREF: Handbuch für die deutschsprachige Version der WHO-Instrumente zur Erfassung von Lebensqualität. Göttingen: Hogrefe 2000.

53. Carver CS, Scheier MF, Weintraub JK. Assessing coping strategies: a theoretically based approach. *J Pers Soc Psychol* 1989;56(2):267-83. doi: 10.1037//0022-3514.56.2.267 [published Online First: 1989/02/01]

54. Muller L, Spitz E. [Multidimensional assessment of coping: validation of the Brief COPE among French population]. *Encephale* 2003;29(6):507-18. [published Online First: 2004/03/19]

55. Knoll N, Rieckmann N, Schwarzer R. Coping as a mediator between personality and stress outcomes: a longitudinal study with cataract surgery patients. *European Journal of Personality* 2005;19(3):229-47. doi: 10.1002/per.546

56. Tedeschi RG, Calhoun LG. The posttraumatic growth inventory: Measuring the positive legacy of trauma. *Journal of Traumatic Stress* 1996;9(3):455-71. doi: 10.1002/jts.2490090305

57. Lelorain S, Bonnaud-Antignac A, Florin A. Long term posttraumatic growth after breast cancer: prevalence, predictors and relationships with psychological health. *J Clin Psychol Med Settings* 2010;17(1):14-22. doi: 10.1007/s10880-009-9183-6 [published Online First: 2010/01/19]

58. Cadell S, Suarez E, Hemsworth D. Reliability and validity of a French version of the posttraumatic growth inventory. *Open Journal of Medical Psychology* 2015;4:53-65. doi: 10.4236/ojmp.2015.42006

59. Maercker A, Langner R. Persönliche Reifung (personal growth) durch Belastungen und Traumata: Validierung zweier deutschsprachiger Fragebogenversionen. [Posttraumatic personal growth: Validation of German versions of 2 questionnaires.]. *Diagnostica* 2001;47(3):153-62. doi: 10.1026/0012-1924.47.3.153

60. Campbell-Sills L, Stein MB. Psychometric analysis and refinement of the Connor-davidson Resilience Scale (CD-RISC): Validation of a 10-item measure of resilience. *J Trauma Stress* 2007;20(6):1019-28. doi: 10.1002/jts.20271 [published Online First: 2007/12/25]

61. Sarubin N, Gutt D, Giegling I, et al. Erste Analyse der psychometrischen Eigenschaften und Struktur der deutschsprachigen 10- und 25-Item Version der Connor-Davidson Resilience Scale (CD-RISC). [First analysis of the 10- and 25-item German version of the Connor-Davidson Resilience Scale (CD-RISC) regarding psychometric properties and components.]. *Zeitschrift für Gesundheitspsychologie* 2015;23(3):112-22. doi: 10.1026/0943-8149/a000142

62. Wang L, Shi Z, Zhang Y, et al. Psychometric properties of the 10-item Connor-Davidson Resilience Scale in Chinese earthquake victims. *Psychiatry Clin Neurosci* 2010;64(5):499-504. doi: 10.1111/j.1440-1819.2010.02130.x [published Online First: 2010/10/07]

63. Notario-Pacheco B, Solera-Martínez M, Serrano-Parra MD, et al. Reliability and validity of the Spanish version of the 10-item Connor-Davidson Resilience Scale (10-item CD-RISC) in young

For peer review only - http://bmjopen.bmj.com/site/about/guidelines.xhtml

adults. *Health Qual Life Outcomes* 2011;9:63. doi: 10.1186/1477-7525-9-63 [published Online First: 2011/08/09]

64. Guihard G, Deumier L, Alliot-Licht B, et al. Psychometric validation of the French version of the Connor-Davidson Resilience Scale. *L'Encéphale* 2018;44(1):40-45. doi: 10.1016/j.encep.2017.06.002

65. Zimet GD, Powell SS, Farley GK, et al. Psychometric characteristics of the Multidimensional Scale of Perceived Social Support. *J Pers Assess* 1990;55(3-4):610-7. doi: 10.1080/00223891.1990.9674095 [published Online First: 1990/01/01]

66. Denis A, Callahan S, Bouvard M. Evaluation of the French version of the multidimensional scale of perceived social support during the postpartum period. *Matern Child Health J* 2015;19(6):1245-51. doi: 10.1007/s10995-014-1630-9 [published Online First: 2014/11/05]

67. Studer J, Baggio S, Dupuis M, et al. Substance use in young Swiss men: The interplay of perceived social support and dispositional characteristics. *Substance Use & Misuse* 2017;52(6):798-810. doi: 10.1080/10826084.2016.1264966

68. Pocnet C, Antonietti J-P, Strippoli M-PF, et al. Individuals' quality of life linked to major life events, perceived social support, and personality traits. *Quality of Life Research* 2016;25(11):2897-908. doi: 10.1007/s11136-016-1296-4

69. Kristensen TS, Hannerz H, Høgh A, et al. The Copenhagen Psychosocial Questionnaire--a tool for the assessment and improvement of the psychosocial work environment. *Scand J Work Environ Health* 2005;31(6):438-49. doi: 10.5271/sjweh.948 [published Online First: 2006/01/24]

70. Dupret É, Bocéréan C, Teherani M, et al. Le COPSOQ : un nouveau questionnaire français d'évaluation des risques psychosociaux. *Santé Publique* 2012;24(3):189-207. doi: 10.3917/spub.123.0189

71. Nübling M, Stößel U, Hasselhorn HM, et al. Measuring psychological stress and strain at work - Evaluation of the COPSOQ Questionnaire in Germany. *Psychosoc Med* 2006;3:Doc05. [published Online First: 2006/01/01]

72. Burr H, Berthelsen H, Moncada S, et al. The Third Version of the Copenhagen Psychosocial Questionnaire. *Safety and Health at Work* 2019;10(4):482-503. doi: https://doi.org/10.1016/j.shaw.2019.10.002

73. Postmes T, Haslam SA, Jans L. A single-item measure of social identification: reliability, validity, and utility. *Br J Soc Psychol* 2013;52(4):597-617. doi: 10.1111/bjso.12006 [published Online First: 2012/11/06]

74. Doosje B, Ellemers N, Spears R. Perceived intragroup variability as a function of group status and identification. *Journal of Experimental Social Psychology* 1995;31(5):410-36. doi: 10.1006/jesp.1995.1018

75. Leach CW, van Zomeren M, Zebel S, et al. Group-level self-definition and self-investment: A hierarchical (multicomponent) model of in-group identification. *Journal of Personality and Social Psychology* 2008;95(1):144-65. doi: 10.1037/0022-3514.95.1.144

76. Aiken LH, Sermeus W, Van den Heede K, et al. Patient safety, satisfaction, and quality of hospital care: cross sectional surveys of nurses and patients in 12 countries in Europe and the United States. *British Medical Journal* 2012;344:1-14. doi: 10.1136/bmj.e1717

77. Aiello A, Khayeri MY, Raja S, et al. Resilience training for hospital workers in anticipation of an influenza pandemic. *J Contin Educ Health Prof* 2011;31(1):15-20. doi: 10.1002/chp.20096 [published Online First: 2011/03/23]

For peer review only - http://bmjopen.bmj.com/site/about/guidelines.xhtml

78. Maunder RG, Leszcz M, Savage D, et al. Applying the lessons of SARS to pandemic influenza: an evidence-based approach to mitigating the stress experienced by healthcare workers. *Can J Public Health* 2008;99(6):486-8. doi: 10.1007/bf03403782 [published Online First: 2009/01/20]

79. Gilles I, Perriraz M, Peytremann-Bridevaux I. Determinants of healtcare professionals well-being at work during a pandemic: a cross sectional study among 10 hospitals in Switzerland. *Manuscript in preparation* 2020

80. Liu Q, Luo D, Haase JE, et al. The experiences of health-care providers during the COVID-19 crisis in China: a qualitative study. *Lancet Glob Health* 2020;8(6):e790-e98. doi: 10.1016/s2214-109x(20)30204-7 [published Online First: 2020/06/24]

81. Tam CW, Pang EP, Lam LC, et al. Severe acute respiratory syndrome (SARS) in Hong Kong in 2003: stress and psychological impact among frontline healthcare workers. *Psychol Med* 2004;34(7):1197-204. doi: 10.1017/s0033291704002247 [published Online First: 2005/02/09]

82. Fiksenbaum L, Marjanovic Z, Greenglass ER, et al. Emotional exhaustion and state anger in nurses who worked during the SARS outbreak: The role of perceived threat and organizational support. *Canadian Journal of Community Mental Health* 2006;25(2):89-103. doi: 10.7870/cjcmh-2006-0015

83. William J. Lancee PD, Robert G. Maunder MD, David S. Goldbloom MD. Prevalence of psychiatric disorders among Toronto hospital workers one to two years after the SARS outbreak. *Psychiatric Services* 2008;59(1):91-95. doi: 10.1176/ps.2008.59.1.91

84. Evanoff BA, Strickland JR, Dale AM, et al. Work-Related and Personal Factors Associated With Mental Well-Being During the COVID-19 Response: Survey of Health Care and Other Workers. *J Med Internet Res* 2020;22(8):e21366. doi: 10.2196/21366 [published Online First: 2020/08/09]

85. Buselli R, Corsi M, Baldanzi S, et al. Professional quality of life and mental health outcomes among health care workers exposed to Sars-Cov-2 (Covid-19). *International Journal of Environmental Research and Public Health* 2020;17(17):6180.

86. Pelz C, Schmitt A, Meis M. Knowledge Mapping als Methode zur Auswertung und Ergebnispräsentation von Fokusgruppen in der Markt-und Evaluationsforschung. *Forum Qualitative Sozialforschung / Forum: Qualitative Social Research* 2004; 5(2). https://nbn-resolving.org/urn:nbn:de:0114-fqs0402351.

87. Burgess-Allen J, Owen-Smith V. Using mind mapping techniques for rapid qualitative data analysis in public participation processes. *Health Expect* 2010;13(4):406-15. doi: 10.1111/j.1369-7625.2010.00594.x [published Online First: 2010/06/17]

88. Trochim W, Kane M. Concept mapping: an introduction to structured conceptualization in health care. *Int J Qual Health Care* 2005;17(3):187-91. doi: 10.1093/intqhc/mzi038 [published Online First: 2005/05/06]

89. Vail EF. Knowledge Mapping: Getting Started with Knowledge Management. *Information Systems Management* 1999;16(4):16-23. doi: 10.1201/1078/43189.16.4.19990901/31199.3

Figure legends:

1. Figure 1: Study's theoretical framework

2. Summary study timeline (March 2021 to September 2023)

For peer review only - http://bmjopen.bmj.com/site/about/guidelines.xhtml

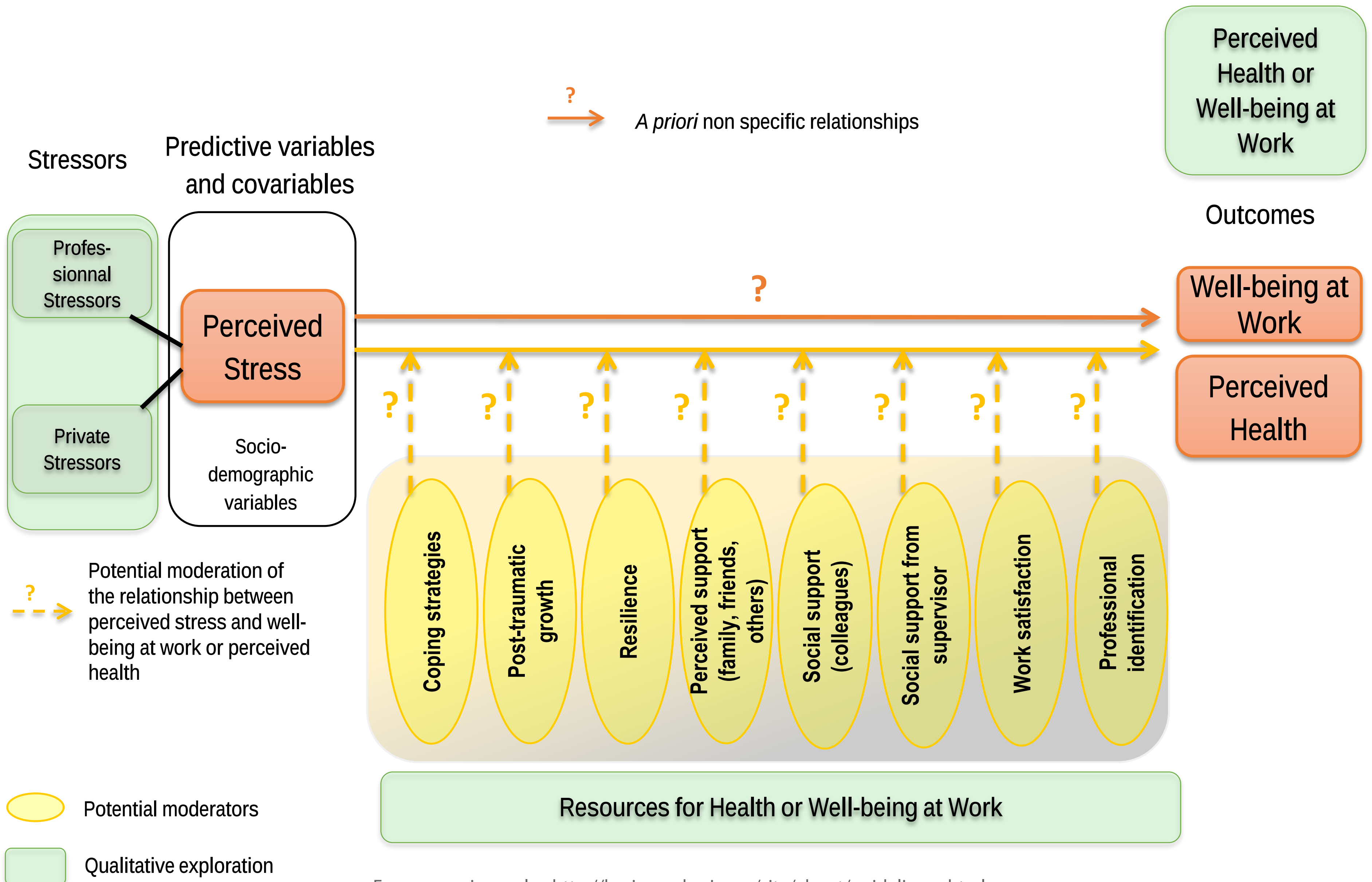

Figure 1: Study's theoretical framework

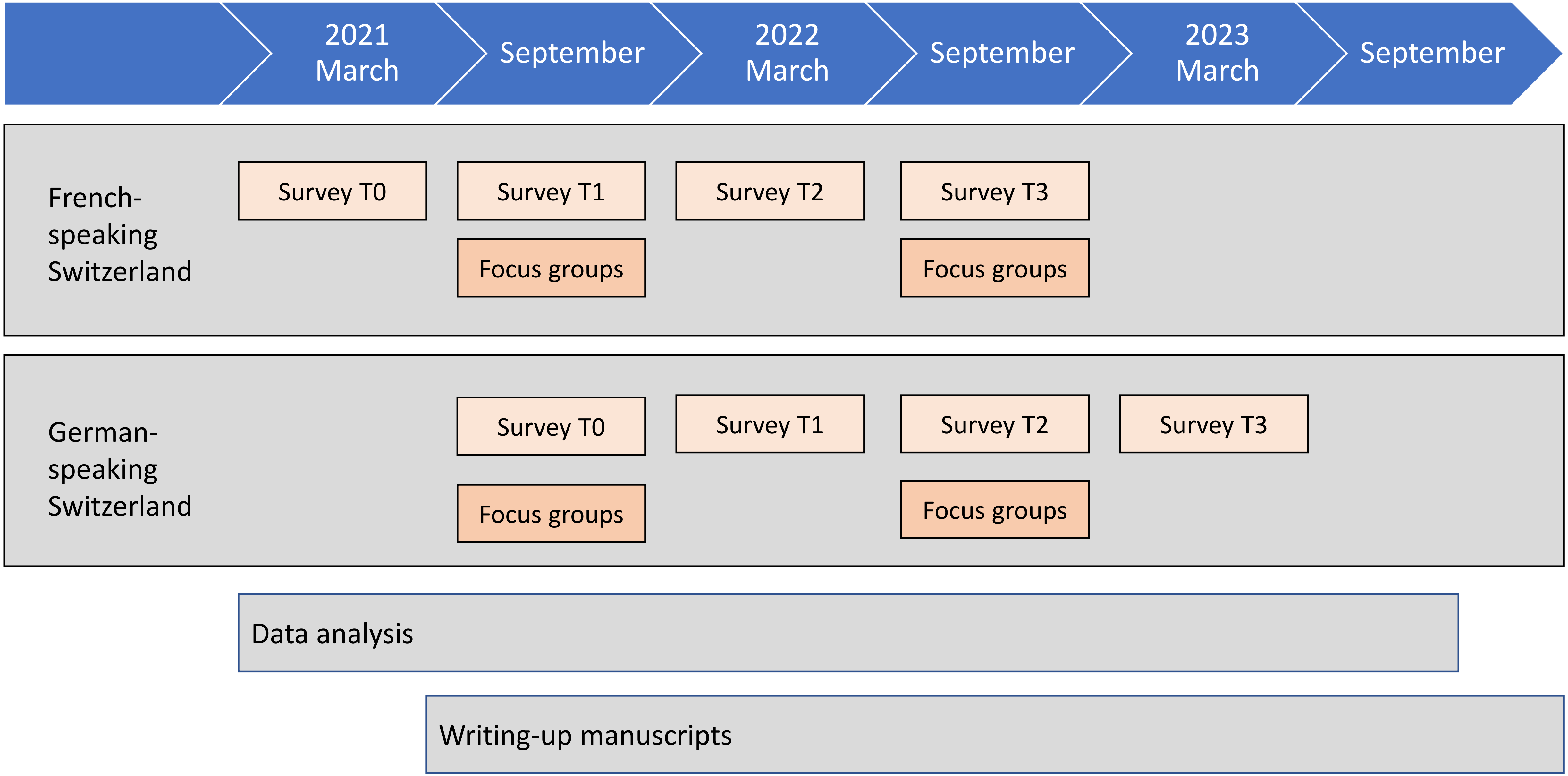

Figure 2: Summary study timeline (March 2021 to September 2023)
